# How can complex interventions be adapted to context? A qualitative analysis of the implementation of DREAMS in three Kenyan settings

**DOI:** 10.1371/journal.pgph.0006701

**Published:** 2026-09-25

**Authors:** Anushé Hassan, Alizée McLorg, Vivienne Kamire, Venetia Baker, Jane Osindo, Elizabeth Kemigisha, Jane Ferguson, Martina Mchenga, Sian Floyd, Daniel Kwaro, Abdhalah Ziraba, Elona Toska, Brendan Maughan-Brown, Isolde Birdthistle

**Affiliations:** 1 Faculty of Epidemiology and Population Health, LSHTM, London, United Kingdom; 2 Kenya Medical Research Institute, Kisumu, Kenya; 3 African Population and Health Research Centre, Nairobi, Kenya; 4 Independent Consultant, Tannay, Switzerland; 5 University of Cape Town, Cape Town, South Africa; Jhpiego, UNITED STATES OF AMERICA

## Abstract

In Kenya, the DREAMS Partnership (“Determined, Resilient, Empowered, AIDS-free, Mentored and Safe”) aims to reduce new HIV cases among adolescent girls and young women (AGYW; aged 10–24) through a comprehensive programme of interventions. The impact of DREAMS varies across settings but reasons for this are not well understood. One potential reason is the differential implementation of DREAMS interventions due to on-the-ground adaptations made by implementers. We sought to understand if, how, and why DREAMS delivery was adapted in three Kenyan settings, so that complex interventions like DREAMS can be tailored to diverse contexts and challenges.We analysed 40 transcripts from individual and group discussions with DREAMS implementing teams. Data were collected between 2017–2019 in rural Gem and two Nairobi informal settlements. Using both FRAME and CFIR frameworks, we identified programming and contextual challenges that affected implementation, local adaptations made in response to these realities and challenges, and the goals and reasoning for adaptation. Reasons for adapting DREAMS were similar across settings yet the type of adaptation varied by context. Adaptations were made to: 1) improve programme attendance; 2) tailor content to better suit AGYW, encourage programme attendance and address community concerns; 3) support participants’ wellbeing, amidst poverty, hunger and violence; and 4) cope with insufficient programming resources. Given their close contact with participants and families, DREAMS mentors often developed unique context-specific strategies to make interventions more appealing and accessible to participants of different ages and circumstances. Understanding shared reasons for adaptation across settings can help anticipate challenges before implementation, and inform the co-design of interventions with communities. As context-specific issues inevitably arise during implementation, mentors can be instrumental in adapting DREAMS, serving as a bridge between programme implementers and beneficiaries. Supporting mentors’ ideas and ongoing commitment can ensure that those delivering programmes plan, learn, adapt and improve in partnership with communities.

## 1. Introduction

In Kenya, HIV incidence among adolescent girls and young women (AGYW) aged 15–24 years is over three times higher than among males of the same age [1–3]. This disparity is rooted in biological, social and economic vulnerabilities that drive HIV infection rates among AGYW, particularly in geographies with high levels of community-wide prevalence.

Advances in HIV-prevention research have identified a range of effective methods for preventing HIV, including biomedical, behavioural, social and structural interventions [4]. It is increasingly accepted that a combination of effective interventions is needed to address the immediate and myriad root causes of HIV risk [5]. Hence, a combination of interventions was endorsed by UNAIDS, with the specific prevention target that 95% of people at risk of HIV use effective combination prevention by 2025. However, less is known about *how* to deliver multi-component prevention packages, particularly for groups at highest risk of acquiring HIV.

The Global AIDS Strategy 2021–2026 [6] and The Global Fund Strategy 2023–2028 [7] support a tailored approach to the implementation of HIV prevention programming at a subnational level. While adapting programmes to local needs and contexts will ensure relevance, little guidance exists to help tailor implementation to boost programme acceptability and success. The DREAMS Partnership (“Determined, Resilient, Empowered, AIDS-free, Mentored and Safe”) was announced in 2014, aiming to reduce HIV incidence among AGYW in more than 150 sub-national units across 10 countries, and subsequently expanded to 15. DREAMS promotes a core package of evidence-based interventions shown to mitigate risk factors for HIV infection [8,9]. This includes the ‘social asset building’ intervention which promotes participants’ networking with peers and female mentors in ‘safe spaces’ (small, female, mentor-led group meetings in safe public places), enhances support, and facilitates links to additional DREAMS interventions and services [9]. In addition, DREAMS offers a unique opportunity to learn from real-world implementation in diverse socio-cultural and epidemiological contexts.

In Kenya, the DREAMS programme has been independently evaluated in three of six DREAMS settings since 2016: Gem sub-county in Siaya County (rural Western Kenya); and two informal settlement areas of Nairobi (Korogocho and Viwandani). Findings demonstrate heterogeneity in the impact of DREAMS by 2019 [10]. For example, in Gem and Nairobi (both Nairobi settlements combined), DREAMS increased knowledge of HIV status, social support and self-efficacy by 2019 [11,12]. However, in Gem but not Nairobi, there was also a reduction in condomless sex and number of lifetime sexual partners among younger AGYW, which was attributable to DREAMS [11]. The reasons for these differential impacts are poorly understood and likely to be highly dependent on context and implementation [13]. The settings differ in terms of culture, demography and geography, as well as infrastructure and availability of schools, resources, income opportunities and existing HIV prevention services. Additionally, in each setting, the core package of DREAMS interventions was delivered by different implementing partners.

In this study, we sought to understand how and why DREAMS was adapted to contextual realities and challenges in three Kenyan settings. Our aim is to learn from the implementation of DREAMS to understand how complex, multi-level intervention can be customised to different geographic, epidemiological, and cultural contexts, for maximum effectiveness.

## 2. Materials and methods

### 2.1. Ethics statement

This study was approved by KEMRI Scientific and Ethics Review Unit for Gem (KEMRI/SERU/CGHR/080/3402), AMREF Ethical and Scientific Review Committee for Nairobi (ESRC P298/2016), and the London School of Hygiene & Tropical Medicine Research Ethics Committee (11835). A research license was also obtained from the National Commission for Science Technology and Innovation (NACOSTI/P/17/69444/15553 for Nairobi). Interviews and discussions were conducted in participants’ chosen language (Luo, Swahili, or English), and transcribed and translated into English. Written informed consent was obtained from all study participants.

### 2.2. Study setting and data

Data for this analysis were collected in the three Kenyan settings described above, where DREAMS was rolled out from 2016: the rural sub-county of Gem in Siaya County, and the informal urban settlements of Korogocho and Viwandani in Nairobi. The three settings are described in detail elsewhere [14–16]. Data collection took place in Gem from 17 January to 13 July 2018 and 9 July to 5 December 2019; and in Nairobi from 2 March to 7 April 2017, 7 September to 8 October 2018 and 2 August to 19 September 2019.

For this analysis, we utilised data collected from semi-structured interviews and focus group discussions with adult individuals directly involved in the implementation of DREAMS, specifically:

- Representatives of implementing partner (IP) organisations delivering the core package of DREAMS interventions in each area. This included program managers, program coordinators, field assistants and field officers from two IPs in Gem and one IP in each of Korogocho and Viwandani.
- Mentors: Young women recruited by the IPs from local communities to recruit participants and facilitate the social asset building intervention, which was delivered in community-based “safe spaces” (in venues such as community and church halls).

In total, 14 key informant interviews (KII) were conducted with implementing partners (8 in Gem, 3 in Korogocho, 3 in Viwandani); 6 KII with mentors (all in Gem), and 20 focus group discussions with mentors (8 in Gem, 6 in Korogocho, 6 in Viwandani). The focus group discussions involved 179 mentors (70 in Gem, 59 in Korogocho and 50 in Viwandani), with a range of 5–15 mentors participating in each discussion. See Table 1 for a detailed breakdown of analysed transcripts. We analysed all 40 transcripts across the three settings and the three study years (2017, 2018 and 2019) to represent views across time and place. Transcripts were analysed by two researchers (AH and AM; see reflexivity statements in the Authors’ Information and Reflexivity Statements section). AH analysed 28 transcripts, and AM analysed 20 transcripts, with eight overlapping, to learn from each other during the coding process and reflect on themes which may have been overlooked.

**Table 1:** Number of transcripts analysed by setting, year, method and participant. FGD = focus group discussion(s); KII = key informant interview(s).

	*Gem*		*Korogocho*			*Viwandani*			*Total*
	*2018*	*2019*	*2017*	*2018*	*2019*	*2017*	*2018*	*2019*	
**Implementing Partners**	4 KII	4 KII	1 KII	1 KII	1 KII	1 KII	1 KII	1 KII	14
**Mentors**	4 KII 4 FGD	2 KII 4 FGD	2 FGD	2 FGD	2 FGD	2 FGD	2 FGD	2 FGD	26
**Total per year**	12	10	3	3	3	3	3	3	
**Total**	22		9			9			40

In all interview transcripts (n = 20), respondents were specifically asked if they had made any changes or adaptations to the DREAMS interventions. The question was most often, but not always, phrased like “Have you made any adaptations or changes to the DREAMS interventions so that it better suits your organization or beneficiaries?” (see S1 Text in Supplementary Information). Themes around adaptation and adaptation impact were also derived from other responses which surfaced in the discussions spontaneously. In all FGD transcripts (n = 20) the respondents were not specifically asked if they made any changes, and adaptations were identified through responses to other questions (e.g., questions around challenges they had faced and how these challenges had been addressed or overcome).

### 2.3. Frameworks used

Two frameworks were used in the analysis and reporting of these data: the expanded Framework for Reporting Adaptations and Modifications (FRAME) and the Consolidated Framework for Implementation Research (CFIR). FRAME is a system for classifying adaptations to evidence-based programs or interventions across various fields and settings [17]. It offers an overarching structure to support research exploring the timing, nature, goals and reasons for adaptations and their potential impact on evidence-based interventions. CFIR provides a detailed outline for identifying factors that facilitate, or act as barriers, to the implementation of interventions across settings [18–20].

During analysis, we found three components of FRAME which were relevant to the data and research question: identifying what adaptations were made; identifying the reasons for making the adaptation, i.e., what was the intended goal for each adaptation; and identifying whether the adaptation was planned/proactive or unplanned/reactive. While we did not have data corresponding to the other FRAME components (which are primarily designed to be utilised when an intervention is ongoing as opposed to post-implementation), the components we used aided in teasing out details of adaptations from the transcripts.

CFIR was useful in summarising and categorising the identified adaptations according to themes we developed during analysis that aligned with pre-defined constructs within its five domains: innovation, outer setting, inner setting, individuals roles and characteristics, and implementation process [21]. It was further helpful for comparing these domains and themes across the three intervention settings. This combined process (using FRAME and CFIR) allowed the development of the ‘reasons for adaptation’ presented in this paper.

### 2.4. Data analysis

Data were analysed in NVivo 13 (version R1, 2020) using inductive, semantic and critical realist approaches within thematic analysis. As such, our coding and theme development was directed by the content of the data (i.e., an inductive approach) and reflected the explicit content of the data (i.e., a semantic approach) [22,23]. Our analysis focused on reporting a reality assumed to be evident in the data (i.e., a critical realist approach) [24,25]. Thus, our themes were not pre-determined, but analytic outputs developed from the coding process and generated through our engagement with the data [26]. The steps we followed during the analysis process were adapted from framework analysis, as this allowed us to generate our own themes, as well as incorporate the existing and validated FRAME and CFIR frameworks [27].

**Fig 1:**
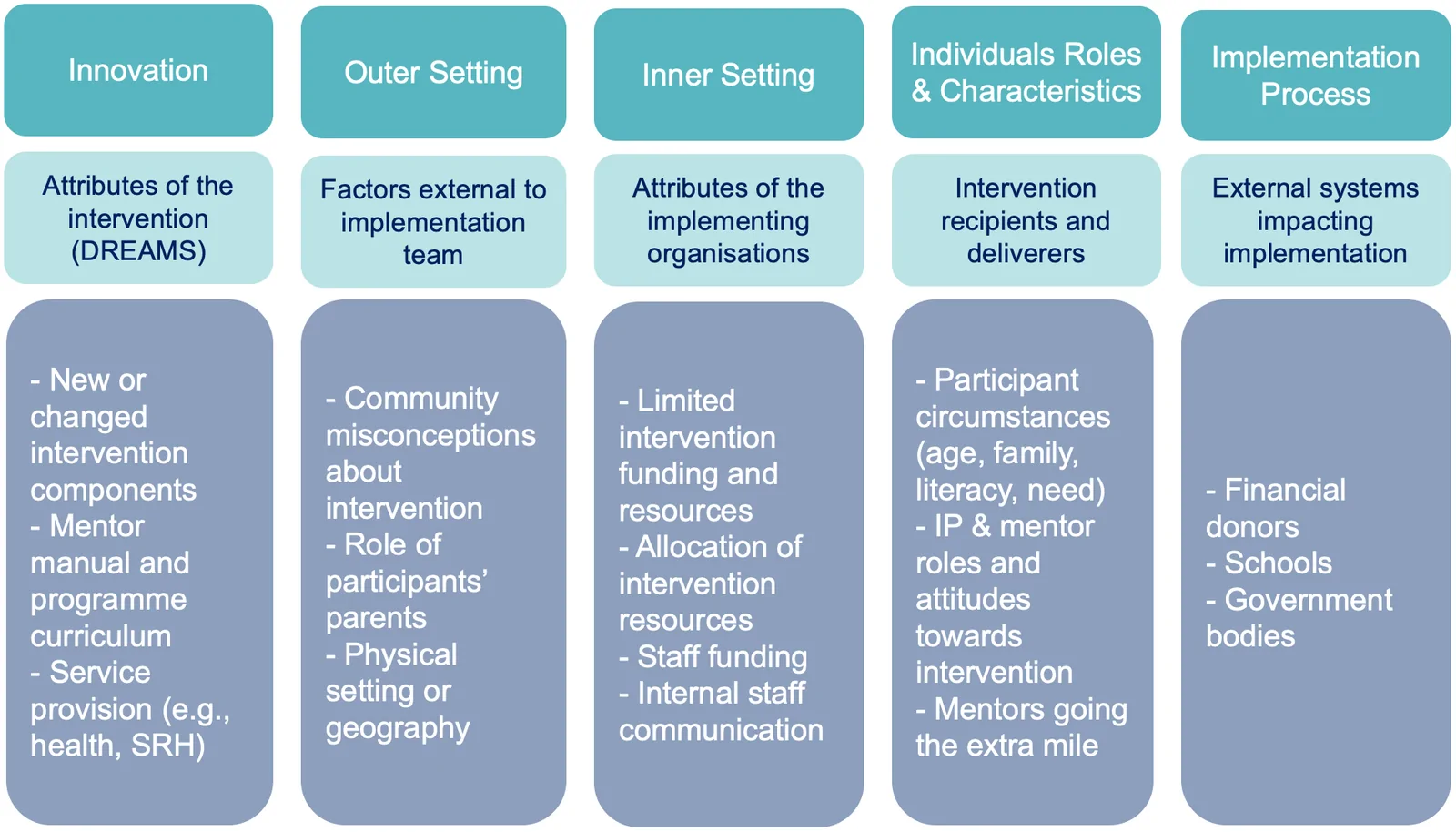
List of themes generated and mapped onto five CFIR domains: Innovation (previously Intervention Characteristics), Outer Setting (external to the intervention), Inner Setting (implementation-related), Individuals Roles and Characteristics (referring to implementors’ and mentors’ attitudes towards the interventions) and Implementation Process. These themes were generated from the factors that influenced the implementation of DREAMS and the contexts that surround each adaptation made to the DREAMS programme.

We first familiarised ourselves with the data by reading through multiple transcripts from different individuals across the three settings (step 1: familiarising). Subsequently, we read each transcript carefully to generate initial themes around adaptation and mapped these onto appropriate CFIR constructs within the five domains (note that the CFIR constructs or domains did not dictate or influence theme generation). For example, we generated a theme “individual circumstances of AGYW” which aligned with the CFIR construct “Patient Needs and Resources” in the “Outer Setting” domain, according to the definition of this construct at the time of analysis (step 2: framework identification). Note that since we conducted our analyses, the CFIR constructs and domains have been revised (see [21]), such that the theme “individual circumstances of AGYW” now aligns with the construct ‘Innovation Recipients’ in the domain ‘Individuals Roles and Characteristics’. This is reflected in Fig 1, which shows the themes we generated mapped onto each of the five CFIR domains, using the updated constructs and domains as described by Damschroder and colleagues in 2022 [21]. We then identified adaptations made to DREAMS that were mentioned by the respondents (the first key component of FRAME used in our analysis). Adaptations were reported by respondents in retrospect and not recorded at the time of adaptation. Full transcripts were reviewed and whenever an adaptation was mentioned by a respondent we coded this as such. Next, we grouped adaptations into appropriate themes, and generated new themes where relevant (step 3: indexing); to do this, we noted any information the respondent provided about the context surrounding the adaptation. For example, one adaptation made by a mentor was to modify the DREAMS curriculum taught to a group of mixed-aged participants. To understand what theme this aligned with, we had to first understand the reason given for this adaptation, i.e., mentors felt the curriculum content was not appropriate for very young girls. As the mentors’ own stated reason for this adaptation was the AGYW’s ages and to improve intervention fit with recipients, we categorised this adaptation within the theme of “individual circumstances of AGYW” (as opposed to “attributes of the DREAMS programme”.) Once we were confident all adaptations had been captured and categorised within NVivo, we created a matrix of the identified adaptations (one adaptation per row) grouped by their corresponding theme and CFIR domain (step 4: charting). The final version of this matrix is provided in S1 Table in Supplementary Information.

A critical realist approach guides the researcher to seek explanations for events or experiences documented in the data by focussing on what individuals can realistically achieve within their social context or setting; it assumes that independent structures facilitate or hinder individuals from pursuing particular actions [25]. Keeping this in mind, we read through the transcripts again to note the respondent’s intended goal for each adaptation where it was possible to identify that goal (the second key component of FRAME used in our analysis) and added the context, intended goal and setting (Gem, Viwandani, Korogocho) for each adaptation to the matrix. We worked back and forth between the matrix and the data to identify any further themes relevant to the adaptations made to the intervention, to contextual factors in the settings, or to the intended goals. Finally, we used the matrix to summarise our findings and comparisons across themes and settings (step 5: mapping and interpretation). This approach enabled us to explore how and why DREAMS was adapted to realities and challenges in different contexts.

## 3. Results

We found numerous factors that influenced adaptations across the three settings. In Fig 1, we depict the most prominent of these, categorised into the five CFIR domains. A detailed matrix in S1 Table illustrates each adaptation identified from the data, categorised within each of the five CFIR domains, along with background information about the intervention or context, the realities and challenges in response to which the adaptation was made, what the intended goal was; and which of the three settings it was identified in. Here, we structure our findings using four overarching themes that featured in all three settings, and refer to these as our main reasons for adaptation. Across all settings, adaptations were made in order to:

1. **Improve** attendance at DREAMS programmes and boost participants’ motivation to attend interventions
2. **Increase** content flexibility in safe spaces
3. **Address** concerns for the well-being of AGYW
4. **Allocate** sufficient resources for all AGYW in need

For each of these reasons, we describe one or more realities or challenges in response to which adaptations were made, describe the adaptation, and, where possible, explain its impact on programme improvements.

### 3.1. Reason 1. The need to improve attendance and boost motivation

Across all settings, IPs and mentors reported struggling to maintain regular participant attendance at ‘safe spaces’ – i.e., safe and private spaces in the community, where mentors delivered DREAMS curricula. IPs and mentors made adaptations to boost participants’ motivation to take part in this intervention component by: making visits to the participants’ homes to encourage attendance; pairing participants as ‘buddies’ to motivate each other to attend safe spaces together; and increasing fun activities at the safe spaces, e.g., introducing soap and detergent making, talent shows, ball games, singing, dancing and debates.

These adaptations were in response to four broad realities or challenges which were negatively impacting attendance and motivation: individual circumstances of AGYW; distrust among parents and the community; perceived exclusion of adolescent boys and young men from DREAMS; and geographical area.

#### 3.1.1. Individual circumstances of adolescent girls and young women (AGYW).

In all three settings, mentors and IPs reported that extra effort was required to boost the participation of older (20–24 years) and out-of-school participants in the safe spaces. This was due to two main reasons. The first was the competing priorities on young women’s time due to their families and children or financial constraints.

*“Sometimes it is hard for those who are age 20 to 24 to attend class … they have children and they are taking them to clinics and others will say they are washing their clothes.”* – **FGD with Mentor, Korogocho, 2018**

The second was because of participants’ male partners resisting their attendance at safe spaces, with some young women facing repercussions at home if they still attended. Mentors stated that male partners often resisted because they thought participation in safe spaces was a waste of the young woman’s time and not helping them contribute anything to the household.

*“Mentor: You realize that most girls like, let me say like from 15 to 24, their husbands don’t want them to come, so talking to them is a challenge and that one is disturbing us. Interviewer: Mmhh, so you have to approach the men? Mentor: Yes, because the women will tell you that if I go, where will I sleep tonight? So, you see we don’t also want them having violent so we have to talk to the men. Sometimes you want to meet the girls and maybe it’s raining, and they have kids, they would not carry their kids, so they end up not attending the mentorship.”* – **FGD with Mentor, Viwandani, 2017**

*“There are barriers with the married girls whose husbands don’t want them to go visit their meeting places … after returning home from the safe space the husband will quarrel with her then.”* – **KII with Mentor, Gem, 2018**

**Adaptations:** To address older participants’ financial constraints, IPs and mentors responded by offering more financial and vocational services. This included money-generating activities like catering skills to redress the trade-off of losing time and money by attending safe spaces; and ‘table banking’, a group funding strategy which allows building of financial assets and skills, where a group of individuals pool together money at specified times during a given period (e.g., monthly), allowing other members to borrow from the pool and return with interest, thus letting the pool grow till the end of the period when the total amount is redistributed among members.

**“***I think mine is a little different because I deal with out of school [participants] more, we do business, we make soap, sell, make profit and currently we borrow loans as we practice table banking. This has kept them busy and serious so they don’t miss safe space because they know very well if they miss someone else will go with the money so we are faring on with table banking.” -* ***FGD with Mentors, Gem, 2019***

To address competing priorities, the IPs spoke with vocational training-schools to introduce babysitting to help AGYW with children, which was successful at one vocational school.

*“… we had challenges with the uptake of these categories of girls - those who head households, those with spouses and those with small children, so basically you find them not enrolling to vocational training because of the limitation that each category has … those with small kids you find they don’t have babysitters or someone they can leave their babies with so in that case we talked to some vocational schools to help with baby-sitting those kids when girls are in class and it has worked well in one vocational school whereby there is a staff for baby-sitting the kids when girls are in class.”* **– KII with IP, Viwandani, 2017**

They also rescheduled safe-space meetings to weekends to accommodate participants who attended school, or reduced the frequency of meetings. Some participants in Gem struggled to understand the content of some of the trainings they were provided due to low literacy levels or language barriers; mentors organised literacy lessons for these participants, and translated program materials into Luo when needed.

To address the resistance participants faced from their partners in attending safe spaces, mentors and IPs held informal meetings with male partners (an ad-hoc meeting not originally in the intervention plan) to let them know about the benefits that DREAMS, and attending safe spaces in particular, would bring to the participant and their households.

*“At the beginning their spouses didn’t want them to come to the safe spaces especially those who got married to the [motorcycle] riders... They thought that their wives will become tough-headed by coming to the safe spaces. But since the introduction of vocational training the men started embracing the participation of their wives and they are now very happy. Even the AGYWs say their sexual partners are now okay with the program.” –* ***KII Mentors, Gem, 2019***

IPs also responded to low attendance at safe spaces by building flexibility into the programme and coming up with entertaining approaches to keep participants motivated and engaged.

*“…we came up with a strategy of how we can improve the social asset building (SAB) uptake, we came up with five pillars for SAB programming which includes ‘AGYW-centric’, meaning … AGYWs choose which income generation activities they want to do in their [safe spaces] and maybe in terms of time and venue and other necessary things required. On top of that is strengthening mentor aspect, involving parents/guardians, making informed decision using data and … strategizing during weekends and holidays.”* **– KII with IP, Viwandani, 2019**

Some mentors visited the participants’ at home to encourage them to attend, others let participants choose the activities they wanted to do during the session and some allowed participants to facilitate sessions.

*“We do follow ups through home visits because we know where they live and we ask them the reasons for missing the class and we encourage them to attend.”* ***– FGD with Mentors, Korogocho, 2018***

*“Sometimes we can give them chance to moderate class on their own and sometimes they can also choose the topic to be discussed in class.”* **– FGD with Mentors, Korogocho, 2018**

**Adaptation impact:** Mentors and IPs in all three settings indicated that programme attendance increased when they initiated the activities mentioned above, which were more suited and appealing to older and out-of-school participants.

#### 3.1.2. Distrust among the community and participants’ parents.

The participation of younger girls (10–19 years) was sometimes affected by parental resistance stemming from community misconceptions about DREAMS and pre-exposure HIV prophylaxis (PrEP), which was new and largely unknown. This included misperceptions that DREAMS was associated with the “illuminati”, alluding to an evil entity or devil worshipping (e.g., DREAMS staff were ‘out to drain people’s blood’), that all DREAMS participants were HIV positive, and that activities in safe spaces encouraged sexual activity and gave out contraception.

*“Initially when we had just started, we were called ‘illuminati” who picks children and ferry abroad…”* **– FGD with Mentors, Gem, 2018**

While DREAMS mentors taught about family planning in their safe spaces and facilitated referrals for contraception if needed, they did not directly provide contraception other than condoms. Mentors reported that some parents also had high expectations and misconceptions about intervention components such as cash transfers and education subsidies, for example, when they saw their neighbours’ children receiving financial benefits they believed that all participants would receive these. They were often discouraged when their own daughters did not receive these benefits, or when their daughters had been receiving cash transfers but stopped receiving them.

*“Some parents may tell their children not to attend classes when they don’t get benefits and they see other people’s children have benefitted with things like school fees …”* **– FGD with Mentors, Korogocho, 2018**

IPs also faced an initial distrust that was created by past programmes in the same areas that had not delivered on their promises.

*“There are even others who refuse to be enrolled but when you think through maybe they were lied to before by another program and another one and it becomes so hard to convince them and there are many CSOs [civil society organisations] that lie, that promise to look for donors but when they go they don’t get the donors.”* **– KII with IP, Korogocho, 2018**

*“So you find that some parents don’t believe in these organisations. They tell you, “others like you also came asking me for money which eventually got lost and now you? I don’t want your help” … I told them that it wasn’t about school fees alone, that there were mentorship programs and things like that but the parents still refuse. “I don’t want those things, you’re just going to use our children because it benefits you and our children will just be left alone.” Such information derails the parents. But if parents can get the right information from the right people I think that can bring more change. So the only thing is how can we organise the parents to get the right information? But so many parents opt out because of bad information.”* **– FGD with Mentors, Viwandani, 2017**

**Adaptations:** Mentors and IPs sensitized the community to the goals of DREAMS by holding extra meetings with parents (sometimes one-on-one home visits). They took time to explain why financial incentives (e.g., cash transfers) were stopped or not included as an intervention, and the importance for participants to continue attending the program to benefit from non-monetary interventions such as social asset building.

*“Our role as mentors is to remind and tell the parents the role of DREAMS - it is not all about financial benefits but there are other important things children learn in classes and is helpful in their lives”* **– FGD with Mentors, Korogocho, 2018**

Some mentors set up feedback boxes, provided informational sessions about PrEP (including for staff such as implementing partners and mentors), or used community radio programmes to overcome community misconceptions.

*“In the mentor groups, we have sensitized AGYW … to demystify issues that come up with PrEP since there are some people who claim it causes infertility or it makes a person to be beautiful … There was a time we were doing so bad in PrEP and we had done everything we knew possible and PrEP was not working for us … Later we realised the solution was to address the myths, misconception and attitudes of staff towards PrEP. We found that we have same attitudes like parents, caregivers, beneficiaries and children towards PrEP. When we addressed this issue of attitude the uptake of PrEP has been well received. We address the issue of attitude through adherence counselling and use of IEC materials where we borrowed some items from CDC and other organizations which have succeeded in provision of PrEP.”* **– KII with IP, Viwandani, 2019**

In Viwandani, a project advisory committee was set up, made up of religious groups, community leaders and AGYW, to get feedback from the community. In Gem, implementing teams responded to negative perceptions due to previous organisations by discussing DREAMS with community chiefs and leaders, while in Nairobi, IPs reported utilising similar methods in conjunction with community radio and SMS systems.

**Adaptation impact:** The main goals were to improve programme delivery, increase access to services and improve programme acceptance. Mentors and IPs reported that parents and community members started valuing and appreciating DREAMS once they saw positive changes resulting from the interventions, such as financial and education support, participant trainings and improved participant behaviour.

*“DREAMS program is expensive, for instance when we involve parents we engage them to hear their perception and you are told of expectations that include allowances, but I tell them there are other benefits that they need to value more than allowances such as change of girls’ behaviour.”* **– KII with IP, Gem, 2019**

This increased motivation to participate in the programme for parents of enrolled AGYW as well as parents of AGYW in the community who were not enrolled in the program. These positive changes were often reported in the second or third year of data collection.

*“By the way, they are very happy because their children have been helped so much by DREAMS. Even those who had refused later came and joined and some are still wishing they could join because when they hear some of the children have been paid fees for, children have been bought uniform or a child has gotten this training which is helpful, they see how good this thing is. Even that issue of illuminati, which first came up, has disappeared, it is no longer there. There is nobody saying their children’s blood will be sucked.”* **– KII with Mentors, Gem, 2019**

On the other hand, in some cases the adaptations – i.e., increased attempts at sensitisation and providing information to parents – were not sufficient to overcome the financial and cultural barriers. In these cases, the parents did not let their daughters enrol onto DREAMS or continue with the program once the financial benefits ended.

*“Many parents in Korogocho only see material benefit from their children coming to DREAMS. They don’t find the knowledge impact important. More awareness should be created on the parents’ part about the importance of the DREAMS package. Sometimes parents realize that their neighbours’ kid is getting material benefit yet their own kid isn’t and word goes around that maybe it is because of how they applied for the project and they end up retracting their children from the project.”* **– FGD with Mentors, Korogocho, 2017**

#### 3.1.3. Perceived exclusion of adolescent boys and young men (ABYM).

Across all three settings, the prioritisation of young women created feelings (among DREAMS participants, parents, young men, and implementing teams themselves) that young men were unfairly excluded from DREAMS interventions. This was despite the provision of some DREAMS interventions for males.

*“The negative effect is that DREAMS focus on AGYW only, so the ABYM feel that they are neglected, and they say that most programs focus on girls so this makes them to feel bad and not being accepted in the community.”* **– FGD with Mentors, Korogocho, 2018**

The perceived exclusion of males was viewed as counterproductive to HIV prevention and female empowerment goals, and a missed opportunity for shifting gender norms.

“*The lack of supporting boys in the program, since you can’t support one gender and leave out the other since the boyfriends of these girls are the boys in the community, for example, if you show a girl to say ‘No’ does the boy also know how to accept the ‘No’. If you go to a family where there are four boys and one girl and they have all dropped out of school and then the girl is taken to school and she is supported with school fees, uniforms, books and sanitary pads while her brothers are at home, this will always bring dysfunction in that family, so if possible I would say that DREAMS project should involve both boys and girls of same age.”* **– KII with IP, Viwandani, 2019**

*“… so, you find it very difficult to tell the boys that actually this program was only meant for girls. So, they see themselves as the inferior in the community. Yeah. So, if the program can allow the boys to be in the program, it can be good.”* **– KII with Mentors, Gem, 2018**

**Adaptations:** Implementing teams increased community sensitization efforts to explain DREAMS’s approach, and why it primarily focussed on AGYW. They also explained that some DREAMS interventions were designed for adolescent boys and young men.

**Adaptation impact:** The increased community sensitization somewhat improved programme acceptance. However, this adaptation had a limited impact. Despite the increased attempts at sensitization and inclusion of more interventions for boys, members of all three communities (in all three intervention rounds, i.e., 2017–2019) said that DREAMS favoured females and excluded males. The adaptations did not help to dispel that perception, due in part to DREAMS’s focus on female HIV risk and also because males did not receive financial support or material resources provided by DREAMS.

#### 3.1.4. Geography.

Geography impacted attendance in DREAMS programmes. For example, Gem, a rural setting, had unique challenges, including inadequate infrastructure for intervention delivery (e.g., to establish safe and private spaces) and long distances with limited transportation between participants’ homes and the locations where DREAMS interventions were implemented.

*“The girls would start a safe space thinking that, that would be the best for them in that region. But maybe because of so many factors around them they feel that it takes a lot of time to walk to that area … maybe in that area there are distractions like maybe loud music; it is close to a road.”* **– KII with IP, Gem, 2018**

In the dense, urban settlement areas of Nairobi, IPs faced challenges ensuring safe and private spaces to deliver interventions.

*“In terms of spaces for meeting … especially for our program, are very limited and even the limited spaces have a cost implication that comes with it so you will find people with halls just hiking their prices arbitrarily and they know anyway there are very few other places that you can go.”* **– KII with IP, Korogocho, 2018**

*“Sometimes you can go to the school and you don’t get vacant class so you will have to teach them on the playing ground and it will be hard for them to concentrate because of noise.”* **– FGD with Mentors, Korogocho, 2019**

**Adaptations**: IPs and mentors in Gem tried to boost accessibility by being flexible and adapting in regards to safe space location.

*“So, at times we change the location to where it is favourable to them.”* **– KII with IP, Gem, 2018**

They introduced additional safe spaces to decrease travel distances for AGYW, and invited HIV-testing counsellors to safe spaces rather than referring participants to health facilities that required travel. Where electricity was not available, IPs used rechargeable solar batteries to ensure that the *MTV Shuga* television episodes and curriculum could be provided in safe spaces. Implementing teams in Nairobi started small libraries for participants as an intervention component; however, when faced with limited space to do this in Korogocho, they instead donated books to an existing library at a partner organisation so participants could go there instead, partnering with other organisations to share space. Multiple mentors in Korogocho reported running their safe spaces in playgrounds when classrooms in the school were not available or too noisy – at times with success and at times not due to distractions and noise in the playground. In both rural and urban settings safe spaces were sometimes merged due to shortage of space or to help mentors share heavy workloads, so the overall number of safe spaces was reduced.

*“Yes, that distance, because they said that place was far that is why I decided to change the safe spaces so that they can also reach here because these two villages they share the two markets. There are people who come from there and come to the market here so at least when I put a central place, they appreciated it … I have not heard any complaint.”* **– KII with Mentors, Gem, 2019**

**Adaptation impact:** While the adaptations improved safe space accessibility and attendance in many cases, not all adaptations were helpful. The merging of safe spaces in Gem reduced the number of safe spaces and led to increased distances for both participants and mentors to travel, which negatively impacted participant attendance. Merging safe spaces in Gem also led to increased workload for the mentors as they were overwhelmed with additional mentees attending each space. Mentors also reported that changing the safe space locations sometimes confused the participants and reduced attendance.

### 3.2. Reason 2. The need for content flexibility

Across all settings, mentors and IP reported that the content and/or delivery of curricula-based interventions needed to be adapted to better suit the AGYW and their communities. Hence, the need for content flexibility was a main driver of adaptation.

**Realities and challenges:** We found that mentors were seen as the main point of contact into DREAMS and, as such, they dealt closely with participants and their families. Because they were also members of the community, they had a good sense of the needs of AGYW and the acceptability of the content among the community. Some mentors (e.g., in Viwandani) reported resistance from parents to sexual education content, such as condom demonstrations and family planning, that they deemed inappropriate, even for 16-year-old girls. Additionally, mentors also had personal views on DREAMS curriculum content, which in some cases influenced what they chose to teach to the participants.

**Adaptations:** With their own understanding of AGYW’s needs and perceptions around acceptability of the content for AGYW, mentors assessed the DREAMS manuals and guidance for local relevance and selected which content to teach. In some cases, implementing teams found the manuals and guidance to be outdated, repetitive, inappropriate or immoral (culturally or due to the participants’ ages). Some mentors reported focussing on new and different activities in the safe spaces – departing from the curriculum content – when participants had gone through the curriculum multiple times and it had become repetitive.

*“Mentor 1: It is not a must we follow a guide to learn sometimes you can ask the girls what they want us to do, and they decide for example to share their life stories or cooking or we can plan a trip. Mentor 2: Sometimes it is not a must to follow manual because it is hectic and we have used it for long so when you know there is a girl who has any skills, for example, skills of making mat she can teach the others how to make and sell those things together as a class”* **– FGD with Mentors, Korogocho, 2018**

A few mentors made modifications to the curriculum when they did not approve of certain topics being taught, such as family planning. While many mentors found the curriculum guided them on which topics to teach to which age groups (e.g., in Korogocho), other mentors (e.g., in Gem) said that because they had one guidebook meant for all ages (10–24 years) some of them had to make decisions about what to teach to each age group.

*“We have one guide that is meant for ages 10 to 24 so it’s upon the mentor to select topics that suits their girls.”* **– FGD with Mentors, Gem, 2019**

On one instance, mentors accidentally taught a session on condom use to the younger aged cohort (10–14 years) when this material was meant for the older aged cohort, due to curriculum for both cohorts being in the same handbook.

**Adaptation impact:** Mentors and IPs adapted the content to encourage AGYW to attend DREAMS programmes and increase acceptance among the communities. Some of these adaptations may not have been consistent with intervention fidelity, if specific mentors were choosing to avoid certain parts of the curriculum – and possibly impacted eventual DREAMS results. It is not clear from our data whether these modifications were monitored for quality and accuracy.

### 3.3. Reason 3. Concern for the well-being of AGYW

DREAMS aimed to enrol AGYW who were considered to be the most vulnerable in the community catchments, many of whom experienced severe hardship in their daily lives, including violence, hunger and poverty. Mentors were selected because of their commitment and passion for improving their communities, and hence mentors expressed genuine care for the participants they worked with. At times, mentors went above and beyond their expected role to care for and support vulnerable participants by giving them more of their time or getting them resources using their own finances.

**Realities and challenges:** When mentors and IPs saw some AGYW struggling, they were emotionally affected and determined to help, even when DREAMS guidance or resources were not available. However, mentors struggled with low pay and inadequate training.

**Adaptations:** While the DREAMS core package includes post-violence care and group-based violence prevention curricula, mentors and IPs introduced private gender-based violence (GBV) sensitization with female participants and their parents or male partners. They also accompanied participants to help them receive GBV clinical and police services.

There were some unintended adaptations likely resulting from a combination of programme failure and the mentors’ compassion for the participants. For examples, at times mentors paid out of their own pocket to provide for AGYW (e.g., food, school uniforms, health service referrals, emergency contraception and pregnancy test kits).

*“Some deserve to be given even the uniform, for example, the girl I was saying … that child used to go to school with torn blouse so I once bought a blouse and went and gave her, she was so happy and the children were telling me she never used to come to school early but now she comes very early.”* **– FGD with Mentors, Gem, 2019**

*“With me, the challenge I got was the day that we were actually paid and there was my DREAMS girl that was going to give birth and she didn’t have anything, so I had to use my money to shop for her, prepare food for her, it wasn’t easy.”* **– FGD with Mentors, Viwandani, 2017**

*“Interviewer: Meaning that of all the people who have contributed voluntarily… from their pockets in order to follow up DREAMS related activities there is none who has been compensated? Mentor: There is none.”* **– FGD with Mentors, Gem, 2018**

When they could not provide help themselves, they raised funds from other participants or in their own social circles.

*“Mentor 1: Sometimes a girl may call you that she is in maternity alone … there was one girl I had to raise funds for her from my friends and even sometimes you may stay with them after they give birth and this type of cases we don’t know how we will report them to the office.*

*Interviewer: So when you get challenges like those ones you cannot come and approach the office to assist you?*

*Mentor 2: It will take long time before they process that money, for example, the same girl who was pregnant she fell down when she was here and I am the one who paid motorcycle to take her home and the office did not help even though they saw it.*

*Mentor 1: There were four DREAMS girls, their houses were burnt and when I reported to the office I was told to write their names … and I thought they will be helped even with blankets but … they told me there is nothing they will be given but it is a must to report those cases. So, I had to ask for contributions from other girls [participants] in the SAB [social asset building] to help them and they were helped”* **– FGD with Mentors, Viwandani, 2018**

In many cases, mentors dedicated their own time outside of work hours, for example, visiting participants at their homes to find out why they weren’t attending safe spaces and encouraging them to attend and picking participants up from their homes to take them to safe spaces.

*“Even if they tell me I don’t put effort, I live in this community and it is a must when a girl misses a class I will visit her and ask her the reason for missing because I took that role to be a mentor although they don’t want to acknowledge it and also they cannot understand this because they are in the office”* **– FGD with Mentors, Viwandani, 2018**

Mentors also organised mentor forums and monthly meetings to exchange tips and share experiences and solutions to these challenges with other mentors. Some of these were self-funded and self-organised, without support from the IPs.

**Adaptation impact:** Mentors made the most of the resources at their disposal, often going above and beyond by using their own funds to ensure participants felt supported and received the necessary care and services. Unfortunately, the programme did not seem to sufficiently adapt to support mentors, exacerbating their financial struggles and adding to their personal and financial burdens.

### 3.4. Reason 4. Insufficient resources

There were three types of resource-based realities and challenges faced by implementors and mentors which led to adaptations. Some participants and families felt they were unfairly excluded from receiving resources as part of the DREAMS intervention and blamed mentors; at times, there were insufficient resources available to the mentors to deliver the intervention as planned; and there were limited resources available at local facilities which the DREAMS implementing teams had to coordinate with to deliver the intervention.

#### 3.4.1. Perceptions of unfair exclusion from receiving resources as part of the intervention.

Material resources provided through DREAMS, including dignity packs (i.e., menstrual health supplies), school subsidies, cash transfers (eventually phased out) and business start-up supplies, were highly valued by participants and their families. These resource and financial interventions were meant for the most vulnerable participants, however there were many DREAMS participants who had considerable needs but because they were not considered the ‘most vulnerable’ they did not receive these interventions. This led to feelings of unfairness and exclusion and mentors were frequently blamed for a perceived bias (some reported harassment and hostility).

*“This you find may discourage some parents or even the children, saying they don’t see how [the IP] is helping them since some people are paid fees for but others are left, some also think it is the mentor who has chosen.”* ***– FGD with Mentors, Gem, 2019***

*“To add on that when girls have missed education subsidy the parents will meet you in the village and ask you why her child was not offered those items. It also leads to enmity because she will assume you are being unfair to her child as you are considering other children”.* ***– FGD with Mentors, Gem, 2019***

IPs and mentors were pressured to provide more resources or to be transparent about allocation decisions.

**Adaptations:** Some allocation issues stemmed from donor targets, with IPs trying to work within these constraints. In many cases, mentors and IPs responded by setting up systems to allocate resources or communicate decisions about provision. For example, in Korogocho, IPs put a list of participants who received education subsidies outside the office to increase transparency.

*“… they [the community] were saying education subsidy is given to this one and not that so what we do is put the list of the people we have paid for outside so anyone who wants to come and check, they can come and check and see whom we have paid for so it is not a process that is swayed by someone, it is a process that is straight”.* **– KII with IP, Korogocho, 2019**

In Gem, the IPs set-up a community advisory board to inform the community that not all participants would receive financial benefits; and set up a vetting committee so decisions about who received education subsidies were decided collectively, and mentors made participants sign attendance or ‘checklists’ in the safe spaces so that those who were active participants would be considered for financial or resource interventions and subsidies.

*“They [education subsidies] are provided as per the target given by the donor, but the challenge we face is the target is low so we develop vetting committees to see which girls should benefit this year but in the previous years, almost 100% of girls got it. We look for the needy of the neediest girls and we vet them for reception of these interventions … the area assistant chief, AGYW representative, school representatives and mentors, they are the ones in charge of pointing out the beneficiaries.”* **– KII with IP, Gem, 2019**

*“Even the parents used to have a feeling that when the girls come for services they need to be paid until it reached a point we could not understand each other so we formed a CAB (community advisory board) that also supported us in sensitization and we had to do meetings for sensitizations so that things could come out clearly and currently the community have understood us.”* **– KII with IP, Gem, 2019**

**Adaptation impact:** We do not have evidence that allocation of tangible resources was perceived as fairer after the efforts to communicate decisions and improve systems for allocating.

#### 3.4.2. Insufficient resources for intervention delivery.

Limited or reduced financial resources were often brought up as negatively impacting implementation. In Gem, a reduction in funding led to a decrease in salary for mentors and some were laid off.

**Adaptations:** In many cases, implementing teams had to improvise with the resources they had available to them. For example, mentors in Gem used flip charts in safe spaces instead of books because no books were given, and made footballs out of polythene bags when the balls they were supposed to receive from the programme were not given, even though participants said they preferred actual footballs.

*“Whenever I go to safe space there’s not even a piece of chalk to write and children prefer that you teach while writing because if you dictate there’s nothing they will get and then we also need to be provided with flip charts and felt pens to use for teaching … At times it forces me to carry some books for the children, I distribute in a manner that one book is split for two children to use”.* **– FGD with Mentors, Gem, 2018**

*“The girls have got sports activities in their safe spaces, we just hear that there are balls for sporting and I have never received any ball in my safe space, and this also includes other safe spaces. Why were the balls bought if they cannot be given to the girls and young women to play with? And if we cannot be given the balls, why can’t we be told the reason why? Because we have been told that the balls are there, who are they planning to give the balls? For us in the safe spaces, we keep on borrowing the balls, we just keep borrowing the balls, we look for our own means of getting the balls.”* **– FGD with Mentors, Gem, 2017**

To adapt to fewer mentors in Gem, some safe spaces had to be combined.

*“We also had issues when we were starting this financial year we merged safe spaces and some mentors had to be laid off due to reduced funding, so some of them were very bitter and were asking girls not to attend safe spaces….The mentors now had to cover long distance to safe spaces so if they came late the mentees could take over some of the activities because each safe space has a mentee.”* **– KII with IP, Gem, 2019**

**Adaptation impact:** Ad hoc supply of extra resources helped to boost participants’ motivation and attendance but demand outstripped supply.

#### 3.4.3. Limited resources at local facilities.

To be appropriately integrated into the community, DREAMS implementing teams had to coordinate with local health services and schools. Some health facilities lacked resources, such as emergency contraceptives, and had limited clinicians available to give services. For some safe spaces that were held in schools, mentors got resistance from the school administration when they arrived to lead safe spaces because the schools had not been communicated with beforehand. Some mentors had official letters from the office to prove their legitimacy, but those who did not have these letters faced difficulties being taken seriously which made implementation of safe spaces more difficult.

**Adaptation:** To compensate for limited resources available at local health facilities, some mentors and IPs paid out of pocket for services to be provided to the participants (e.g., emergency contraceptives). Some IPs in Gem conducted what they referred to as ‘continuous medical education’ for IPs and mentors, which refreshed their knowledge about the DREAMS interventions and trained them to deliver biomedical talks to the participants.

*“When it comes to biomedical, initially we had clinicians with the talks but later we had to do CMEs [continuous medical education; it’s a medical term that medics use so we borrowed it] so that we could all reach the girls at the safe spaces with the information that’s required. We also had to conduct CMEs with the mentors so that they were aware of all the interventions that had been rolled out. Though they had been trained and made aware of them we wanted a situation whereby they could courageously talk about them.”* **– KII with IP, Gem, 2019**

IPs in Viwandani created a referral directory of health facilities where they could refer participants in need, to streamline the referral process. They also formalised local partnerships by developing a memorandum of understanding with these facilities such that they could invoice the implementing organisation for any tests or care, so that participants didn’t need to pay for services. In Korogocho, the IP made extra efforts to learn about different organisations they could refer participants to who had specific needs, and which of these would be the most efficient for participants as well as logistically compatible with the program.

*“…something like shelter, something like legal support is not our strength and is also not part of our core mandate, you know organizations that their entire work is legal support for survivors of gender based violence … maybe this dispensary is very good at providing family planning services and … they do not lack supplies, so once you know what they are good at you go the extra mile of knowing how they operate, if I refer and say from here will they be given preferential treatment, what are their working hours, do they have any charges, and if there are charges do they resonate with the people we work with, so our referrals are usually very targeted.”* **– KII with IP, Korogocho, 2018**

**Adaptation impact:** Providing ‘continuous medical education’ to IPs and mentors was done to reduce the demand on the health system (by task shifting). Improved understanding of and relationships with health facilities in Nairobi was done to streamline referrals, improve the experience for participants, and make it easier and simpler for new staff to make referrals.

*“Yes, every facility has their own referral directory with the name of the facility and the contact person and their mobile phone number and the services they offer so when you come, even if you are new, and you want to refer for these services you will know where and who to call since we have talked to those organizations and they know us.”* **– KII with IP, Viwandani, 2019**

**Fig 2:**
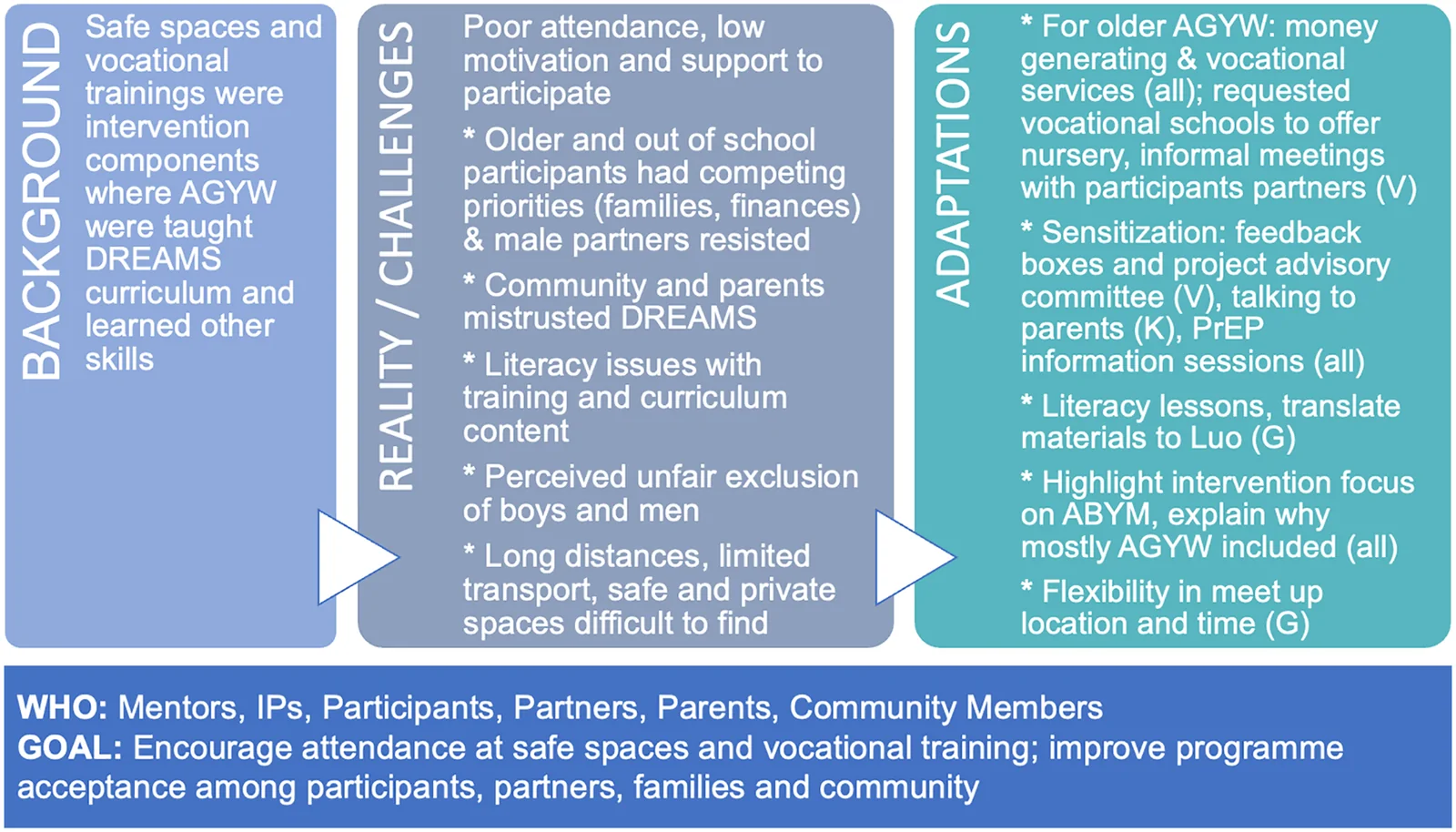
Using the theme ‘individual circumstances of AGYW’ as an example, the figure depicts a summary of what the intervention background was; what the reality or challenges were that led to adaptations; what adaptations were made in each setting; who was involved in the adaptation; and what the goal of the adaptations was. All = all three settings; G = Gem; K = Korogocho; V = Viwandani.

Fig 2 provides an example of how there were similar contextual challenges and drivers that led to adaptations across settings, but the adaptations made by IPs and mentors differed between the three settings.

## 4. Discussion

While globally funded, a complex program like DREAMS is inevitably influenced by local realities, values, and needs, and successful implementation requires adaptation to local conditions. In this analysis of qualitative data from Kenya, we examined adaptations to DREAMS programmes in three different contexts to understand how and why implementation varied. We identified some drivers of adaptation that were setting-specific. For example, differences in geography, infrastructure, literacy, and culture across urban and rural settings posed distinct challenges and necessitated different adaptations to enhance programme reach and acceptability. Other drivers were common across all settings, and IPs and mentors responded in different ways to address them.

Mentors, who were recruited from the same communities as the participants, may have been closer to participants and more aware of their day-to-day challenges. They sometimes expressed greater affinity with the participants than with programme funders. Implementing partners, on the other hand, tended to be more involved with programme structure and funder requirements, and had more direct contact with funders. Although our sample size was limited, these differences in roles may have influenced perspectives and priorities, with mentors often focusing more on participant needs and IPs on programme delivery and targets.

Our results suggest four key ways in which implementation varied across the three contexts analysed: programme attendance, community acceptance of DREAMS, utilising of mentors in programming decision, and resource allocation. Below, we reflect on each of these, followed by a discussion on the importance of programmes like DREAMS to be flexible, and we conclude with some limitations of this study.

### 4.1. Variation in implementation: Programme attendance

We found it was difficult in all settings to maintain regular attendance at DREAMS programmes, particularly for young women aged 20–24 years. This is supported by other studies showing that young women who are out of school have other priorities and pressures that serve as barriers to attending empowerment groups like safe spaces [28]. Furthermore, young women with children may have economic hardship, and are likely to prioritize activities that have a financial incentive [28]. For some participants, attendance was also restricted by their partners. Some partners disapproved of female-centred programming like DREAMS (particularly the sessions held in private spaces), and when participants attended these sessions they faced serious repercussions at home – this escalated from being an issue of attendance to an issue of safety. Therefore, in the case of DREAMS, adapting safe space curriculum by initiating money-generating activities that women could utilise in their own time was an effective way of incentivizing participants with competing priorities to attend safe spaces. Our findings suggest that programme creativity and incentives - including non-financial ones such as mentoring, social asset building and skills development - provided in safe spaces are necessary to increase and retain attendance in DREAMS programmes. Adaptations such as the ‘continuous medical education’ led by IPs which enabled IPs and mentors to learn and deliver material usually undertaken by clinicians, could become a regular component to promote attendance, helping overcome issues of distance and partner resistance.

Equally, ensuring that implementing teams have sufficient support and time to plan and initiate activities is important, particularly in resource-constrained contexts where non-financial incentives can go a long way. This aligns with some other studies which found that implementers adapted their programmes to be more interactive and utilised visual demonstrations to improve contextual fit and increase participant retention [29–32].

### 4.2. Variation in implementation: Community acceptance of DREAMS

While DREAMS aimed to deliver a package of interventions that involved male partners and community stakeholders, it was widely perceived (by IPs and mentors as well as community members in all settings) to have minimal to no inclusion of males. Most informants in this study stated that this was counter-productive to DREAMS’s aims of gender equity. Excluding ABYM from curriculum- and mentor-based interventions in other DREAMS implementation sites like South Africa has been reported as being inequitable, counterproductive and a missed opportunity for shifting gender norms [33–35]. Evidence from Northern Uganda found that a ‘quality improvement behaviour change’ model was implemented using quality improvement teams under DREAMS. The teams involved male partners of AGYW in meetings and discussed issues around domestic violence, high risk sex and social/financial empowerment of their female partners [36]. Through increased male engagement, females engaged significantly less in transactional sex and experienced less gender-based violence [36].

Our findings also show that community sensitization was necessary to increase acceptability of DREAMS by local communities. In Gem, in particular, the implementing partners (including field staff) alluded to initial community distrust, but repeated conversations and sensitization garnered high community approval and appreciation for DREAMS. These efforts were often in response to challenges, suggesting that investing in upfront (prior to intervention roll-out), contextually appropriate, and targeted community sensitization could be beneficial when implementing DREAMS in other settings. This could result in community ownership of the programme from its very beginning, and also avoid subsequent issues around which AGYW are enrolled and why. Demonstrating that the programme leads to results valued by the community is also important to maintain community support over time.

### 4.3. Variation in implementation: Utilising mentors in programming decisions

It is clear from this analysis, and other analyses with DREAMS mentors in Kenya, that mentors have high knowledge of the communities and beneficiaries of DREAMS and were a key component to the success of DREAMS [37]. They were creative and pragmatic in boosting programme acceptance and attendance and went beyond expectations to ensure the most vulnerable participants were supported. However, their additional efforts could exacerbate personal and financial struggles they already faced (e.g., dedicating their personal time and funds to participants). Mentors reported they were sometimes held responsible by participants’ parents and partners for programme decisions they had not made, while at the same time they were not listened to ‘by their seniors’ (in the office) or allowed to be part of official decision-making. Future implementation of these interventions should ensure that the programme either has sufficient resources to meet participants’ needs without burdening mentors, or – given the widespread funding reductions currently affecting such programmes – de-prioritise resource-intensive components in favour of skill-building activities that foster participants’ financial independence from mentors.

As young women from the same communities as the participants, mentors could be utilised more in decisions and strategies for adaptation, helping to ensure programme strategies take account of community views. Allowing mentors agency to make strategic decisions to adapt the programme could help to embrace and expand the key role they play in adapting DREAMS to local contexts. Their time and dedication would need to be adequately recognised and compensated. Formalising community-led approaches like this could improve programme uptake and acceptance, challenges that were experienced in each setting [38–40]. An example of such a flexible model is the Coach Mpilo programme, a peer mentor project where men newly diagnosed with HIV or experiencing treatment challenges were invited to receive individual coaching from peer supporters or ‘coaches’ who were also living with HIV but were stable on treatment [41]. The coaches were given agency to tailor the programme to the participants’ needs. Offering mentors non-financial incentives such as opportunities for formal qualifications (e.g., similar to the ‘continuous medical education’ initiated by IPs and mentors) could be an additional way to support and acknowledge them.

### 4.4. Variation in implementation: Resource allocation

Lastly, limited resources were reported in all settings, which made implementation more challenging and led to context-specific adaptations. The findings indicate that, to overcome consistent challenges to participant attendance and community acceptability, up-front investments in community sensitization, programme content and flexibility, and support for mentors and field staff, could pay off in the longer term.

### 4.5. Importance of programme flexibility

In regard to the third FRAME component addressed in this study, i.e., whether adaptations made to the intervention were planned/proactive or unplanned/reactive, we interpret all adapations as having been made in an unplanned and reactive manner.

It is in fact unlikely for real world interventions to be implemented exactly as planned: context-influenced adaptations are unavoidable, which may or may not adhere to intervention fidelity, and may or may not impact intervention outcomes. Ensuring that intervention programming adapts to local circumstances without compromising intervention effectiveness is important. It is thus also important to understand and monitor such adaptations for programmes like DREAMS which aim to deliver important sexual health education to adolescents and young women. In line with this, the DREAMS programme has utilised scientific literature and data collected with participants and program staff to make real-time program improvements both in its earlier stages [15] and since [42]. For example, in 2018, in response to information gathered through field observations, and feedback from PEPFAR country teams and intervention participants, PEPFAR instated new requirements that a participant’s receipt of required services had to be recorded (through staff accompaniment or follow-up). Prior to this, many participants had not been accessing the primary interventions required as part of the DREAMS program, and there was no official tracking of how many interventions participants were completing [42]. In Zambia, implementation challenges that were identified early in the program’s lifecycle were addressed through adaptive strategies, for example, setting up in-person coordination meetings for IPs to resolve staff jurisdictional disputes over participant target setting and recruitment [43]. Rosen et al. [43] also conclude that delivering multisectoral interventions such as DREAMS while ensuring intervention fidelity requires a robust implementation infrastructure, including, for example, adaptable workplans.

The Medical Research Council's (MRC) framework for developing and evaluating complex interventions sets out ‘context’ as one of six core elements to reflect on throughout the research process, including during intervention implementation [13]. In this paper we show how implementation of the DREAMS intervention interacted with local context in three Kenyan settings and led to a number of adaptations. Our findings emphasize the need for complex interventions to be planned together with host communities, to be flexible during implementation, and to be led by local stakeholders like IPs and mentors. The MRC framework suggests building flexibility into the intervention as early as the design-stage to allow implementation of some components to vary by context while maintaining ‘the integrity of the core intervention components’ by stating which components do not have room for flexibility during delivery [13]. A contrasting conception of fidelity has also been proposed, namely ‘functional fidelity’, which critiques focussing on an intervetion’s ‘core components’, instead suggesting that intervention activities can be adapted and/or substituted as long as the same theory of change is valid in the new context or circumstance [44].

We do not have evidence (from the data analysed) on how the adaptations identified in this paper directly influenced DREAMS outcomes, if they interfered with the integrity and effectiveness of the core DREAMS intervention components, or whether the same theory of change was activated in new contexts. For example, modification of DREAMS curriculum content by mentors, and how they chose to deliver this content, may have affected the accuracy and completeness of information designed to help AGYW prevent HIV. We cannot tell from the transcripts whether any adaptations were formally monitored. A framework like FRAME offers a tool to manage and monitor adaptations during intervention delivery. For programmes like DREAMS which are implemented across numerous settings, co-designing interventions with local stakeholders can help implementing teams anticipate delivery challenges that may require flexibility. Incorporating flexibility during the design stage can help reduce challenges teams are not prepared for; and methodically monitoring adaptations can help ascertain associations between adaptations and outcomes [13,17].

The development of a formal system that not only monitors adaptions but also anticipates, develops, and manages them could be quite beneficial. The conception of different approaches to deal with challenges faced during DREAMS intervention delivery in different settings also underscores the need for this system to facilitate sharing of ideas across locations. A system that clarified to IPs and mentors the need to raise potential areas that could be improved with adaptations would convey that adaptations were in fact valued by senior programme staff. In turn, this could help overcome some challenges and tensions faced by mentors who said the programme took a toll on their personal and financial lives while they were also held accountable for decisions made by senior programme staff. This would further create buy-in from beneficiaries and their family members, as well as other programme staff who would understand that any adaptations or changes made were a programme decision based on participant feedback and mentors’ insights – and not mentors making rogue or self-interested decisions. It is equally important for the programme to highlight adaptations that may have adverse effects. For example, some mentors adapted the safe space curriculum content by removing content which they thought was not suitable for moral reasons (e.g., family planning topics). However, removing such content could deny participants the information, skills or motivation they need to make their own decisions.

### 4.6. Limitations

Our study has some limitations. First, there was an imbalance between data available for Gem and the two Nairobi settlements. Data from Nairobi are available over a longer time period (2017–19, compared to Gem where data were not collected in 2017), however, fewer interviews and focus groups were conducted per year in Nairobi than in Gem. While this led to more in-depth information about Gem than the two Nairobi settlements, there was less information available about earlier challenges and adaptations in Gem (in 2017). In Korogocho and Viwandani, only one interview per year was conducted with implementing partners, as opposed to Gem, where there were two interviews with each of two implementing partners each year. Thus, we may have picked up on a wider variety of adapations from Gem than from the two Nairobi settings. Similarly, no interviews were conducted with mentors in Nairobi (only focus groups) which led to richer information for Gem where both interviews and focus groups were conducted with mentors.

Second, while we comment on community and beneficiary perspectives in this paper, we acknowledge that those perspectives are presented by IPs and mentors (as experienced by themselves) and not from the community members and beneficiaries themselves. Also, while the interview questions were similar across all settings, it was at times difficult to identify adaptations from these transcripts comprehensively, because IPs and mentors may not have mentioned each and every adaptation that they made. While this analysis benefited from an extensive series of in-depth interviews conducted over time in three distinct settings, we did not have access to programme data and are not aware if adaptations were routinely or consistently monitored at programme level. Nor did we have consistent data on the impact of adaptations made. We relied on implementers’ retrospective self-reports of challenges they experienced and adaptations they made in response. Going forward, learning opportunities could be enhanced if DREAMS implementing teams documented programme adaptations, for example, using the FRAME tool. This information could also enable an analysis of how heterogeneity in DREAMS implementation influenced its impacts, e.g., on young women’s sexual and reproductive health outcomes across settings.

## 5. Conclusion

To address the multiple causes of complex health challenges, multi-component interventions are needed. In this analysis, we aimed to understand how and why DREAMS, a complex intervention for HIV prevention, was adapted to local context in three Kenyan settings. We found that across all settings adaptations were needed to address shared challenges: to improve programme acceptance; to adapt content to better suit the participants; to address community concerns; to better support the welfare of participants; and to cope with limited program resources. Programmes like DREAMS can anticipate these challenges and plan locally-specific adaptations in close collaboration with communities. As new challenges inevitably arise during implementation, implementers should have the flexibility to adapt in ways that meet local needs without jeopardising the integrity of the core intervention. This can save time and resources, and build community ownership. We found that mentors are uniquely placed to act as a bridge between the community and the programme; they understood and responded to participant needs and should be supported (financially and professionally) to continuously improve programming.

## Supporting information

S1 Text Sample topic guides used to interview implementing partners. (DOCX)

S1 Table List of adaptations with supporting information. (DOCX)

## Reflexivity statements from authors who analysed the data

Anushé Hassan (AH): AH was working as Research Fellow at the London School of Hygiene and Tropical Medicine (LSHTM) when this paper was written. She grew up in Pakistan, undertook her undergraduate and postgraduate education in the UK, and has experience in conducting research in East Africa, but not in Kenya. She is comfortable discussing topics around sexual and reproductive health (SRH), reproductive rights and justice. Her views on HIV and SRH may differ from those held by some of the participants of this study, and she does not have direct experience or personal knowledge of the contexts where this work was undertaken. This likely influenced the lens through which she interpreted the data. She acknowledges that it is impossible to completely disentangle her own perspectives and positionality from the participants’ narratives and this may have led to some bias in her interpretation of the data, and thus also in theme generation and categorisation of adaptations within themes.

Alizée McLorg (AM): At the time of analysis, AM was a Master of Science Student in Demography and Health at the London School of Hygiene and Tropical Medicine (LSHTM). Before studying at LSHTM, AM worked in health services research focusing on patient engagement in the United States. AM’s views on HIV and adolescent sexual and reproductive health (SRH) have been influenced by her upbringing and education in public health, and, as such, she is comfortable with discussing SRH. As an outsider to the context, she will likely have missed contextual nuances mentioned in the transcripts and likely has differing views on HIV and sexual and reproductive health from the implementing teams. However, AM has previous experience studying and working in Nairobi and Western Kenya, which helped her to picture the context. This previous experience also introduces bias, likely in trying to confirm her pre-existing views of Nairobi and Western Kenya. She tried to mitigate this by only interpreting contextual factors with the information provided and omitting her previous ideas of the context.
