# Supplementary material for: How can complex interventions be adapted to context? A qualitative analysis of the implementation of DREAMS in three Kenyan settings
Source: PLOS Glob Public Health. 2026 Sep 25;6(9):e0006701. doi: 10.1371/journal.pgph.0006701 (PMC13614640; doi:10.1371/journal.pgph.0006701)
Supplement: Supplementary file 1 [file pgph.0006701.s001.docx]

**S1 Text. Sample topic guides used to interview implementing partners**

# *Key informant interviews with Implementing Partners - 2018 and 2019*

The questions below are a guide. Use the interview as an opportunity to explore any particular issues or events that may affect the implementation of the DREAMS intervention, that may be barriers and facilitators to implementation, or that capture staff members’ and beneficiaries’ perceptions and value of the intervention.

Complete the cover sheet before beginning the interview (Annex 1).

# Questions/Themes:

1. *(Ask only if participant is a new individual*): How would you describe DREAMS to someone who had never heard about it?

*(Ask previous participant- if new participant, rephrase to reflect current discussion)* Apart from what we discussed previously, what new DREAMS activities is your organisation currently involved in?

2. Please describe your experience of working in Korogocho/Viwandani thus far.
3. Have you experience any challenges in this final year of implementation of the program? If yes, please tell me about them. How did you overcome these challenges?
4. Please tell me about any new or additional work that you/your organisation does since becoming involved with DREAMS.
5. DREAMS initiative comprises different interventions. Can you tell me about your experiences implementing the different components of DREAMS? Do you feel that it has been easier to implement some of the components compared to others? Why?
6. In your view, how has DREAMS been experienced by those who deliver it? (*Refer to link facilities and probe for delivery of HTS, VMMC, ART support groups)* How has it been experienced by the wider community members?
7. How would you describe layering of interventions for the beneficiaries to someone who has never heard of it?
8. How much are ABYM involved in the program until now?
9. How do beneficiaries perceive and value the various DREAMS interventions? What do they think of the quality of the interventions delivered?
10. What measures do you have in place to handle situations of distress that you come across (e.g., GBV, child labor, sexual exploitation etc.) How effective are these measures? What can help to improve the effectiveness of the existing measures?
11. What does success in implementing DREAMS look like? What kinds of things are helping you to move in that direction, and what kinds of things get in the way?
12. How does the reality of DREAMS compare to your expectations
13. Have you made any adaptations or changes to the DREAMS interventions so that it better suits your organisation or beneficiaries? Please tell me about any innovations or changes that you have made to the intervention.
14. In your view, have there been any changes in the communities you work as result of the implementation of the DREAMS initiative? In what ways have they changed and why? What positive changes have there been? What negative changes have there been?
15. What interventions do you feel are impacting the community most? In what way are they impacting the community? Why do you say so? What about those impacting the community the least? Why do you say so?
16. So far, how do your staff perceive and value the various DREAMS interventions? What do they think of the quality of the interventions delivered? How would you say DREAMS is affecting the workload of your staff?
17. What are the main opportunities that you/your organisation has experienced since the beginning of DREAMS?
18. What factors help/facilitate your DREAMS work?
19. What factors hinder/act as barriers to your DREAMS work?
20. What recommendations do you have for improving the DREAMS intervention?
21. What do you think will happen to the work you currently do following the completion of the DREAMS intervention?

Closing

We have come to the end of the interview. Before we finish, is there anything else that you would like to add about what we have discussed?

# *Key informant interviews with Implementing Partners - 2017*

The questions below are a guide. Use the interview as an opportunity to explore any issues or events that may affect the implementation of the DREAMS intervention, that may be barriers and facilitators to implementation, or that capture staff members’ and beneficiaries’ perceptions and value of the intervention.

Complete the cover sheet before beginning the interview (Annex 1).

# Questions/Themes:

1. How would you describe DREAMS to someone who had never heard about it?
2. What DREAMS-related activities is your organisation involved in? What is your specific role on the project?
3. What activities did your organisation conduct before DREAMS? Who were your beneficiaries? How were you funded?
4. What were your expectations of DREAMS before you started?
5. When did the roll-out of the DREAMS activities start?
6. What preparation work was done before the roll-out?
7. Please tell me about any new or additional work that you/your organisation does since becoming involved with DREAMS.
8. What proportion of your time do you spend on DREAMS-related and non-DREAMS-related work?
9. Did you experience any challenges as you were rolling out the program? If yes, please tell me about them
10. I understand your organisation is working in several villages. Please tell me about your experience of working in these villages. Do you feel that it has been easier to implement your work in some of these areas compared to others? Why do you think this may be?
11. The DREAMS initiative comprises different interventions. Can you tell me about your experiences implementing these components? Do you feel that it has been easier to implement your work in some of these areas compared to others? Why?
12. In your view, how is DREAMS experienced by those who deliver it and wider community members?
13. How do beneficiaries perceive and value the various DREAMS interventions? What do they think of the quality of the interventions delivered?
14. What does success in implementing DREAMS look like? What kinds of things are helping you to move in that direction, and what kinds of things get in the way?
15. How does the reality of DREAMS compare to your expectations when you were first starting out?
16. Have you made any adaptations or changes to the DREAMS interventions so that it better suits your organisation or beneficiaries? Please tell me about any innovations or changes that you have made to the intervention.
17. In your view, have there been any changes in the communities you work in since you started implementing the DREAMS initiative? In what ways have they changed and why? What positive changes have there been? What negative changes have there been?
18. How do staff perceive and value the various DREAMS interventions? What do they think of the quality of the interventions delivered? How would you say DREAMS is affecting the workload of your staff?
19. What are the main opportunities that you/your organisation has experienced since the beginning of DREAMS?
20. What factors help/facilitate your DREAMS-related work?
21. What factors hinder/act as barriers to your DREAMS-related work
22. What recommendations do you have for improving the DREAMS intervention?
23. What do you think will happen to the work you currently do following the completion of the DREAMS intervention?
