# Supplementary material for: How can complex interventions be adapted to context? A qualitative analysis of the implementation of DREAMS in three Kenyan settings
Source: PLOS Glob Public Health. 2026 Sep 25;6(9):e0006701. doi: 10.1371/journal.pgph.0006701 (PMC13614640; doi:10.1371/journal.pgph.0006701)
Supplement: Supplementary file 2 [file pgph.0006701.s002.docx]

**S1 Table. List of adaptations with supporting information**

| *Each adaptation identified from the data is categorised below within the five CFIR domains, along with background information about the intervention or context; the reality or challenge in response to which the adaptation was made; what the intended goal of the adaptation was; and which of the three settings it was identified in.* | | | | | |
| --- | --- | --- | --- | --- | --- |
|  | **Background information** | **Reality or challenge in response to which adaptation was made** | **Adaptation** | **Goal** | **Setting** |
| **1** | **CFIR domain: Innovation (previously Intervention Characteristics)** | | | | |
| **1A** | **Attributes of the DREAMS programme that impacted implementation** | | | | |
|  | *As part of the DREAMS programme, some AGYW were selected to receive additional benefits and some were not | *There were not enough 'benefits' (e.g., dignity packs, education subsidies, cash transfers etc.) for all AGYW - not all participants received these benefits *AGYW not receiving these benefits felt excluded or discouraged which impacted their safe space attendance *When cash transfers were reduced it decreased AGYW incentive to attend safe spaces & social asset building (V) *Created frustration for AGYW and their parents (V) | *Mentors gave benefits to more AGYW where possible, e.g., uniforms, bags, dignity packs *Mentors created a vetting committee to involve the community in decisions around who received education subsidies (G) *Mentors felt the need to create an incentive to attend safe spaces and initiated engaging activities in these sessions (G) *Mentors and IPs implemented activities such as cake making, table banking, soap making in the safe spaces (V) | *Increase/encourage attendance of AGYW at safe spaces *Increase AGYW retention in the programme *To motivate and satisfy AGYW *Increase programme acceptance | *Gem (2018, 2019) *Viwandani |
|  | *As part of DREAMS, some AGYW were selected to receive additional benefits or incentives and some were not | *Some AGYW felt excluded | *Mentors created checklists of AGYW to help decide who should receive benefits and school fees etc. | *To ensure benefits were given to AGYW in need | *Gem 2019 |
|  | *As part of DREAMS, some AGYW were selected to receive additional benefits or incentives and some were not | *AGYW used to receive cash transfers and start-up capital as part of the intervention in the earlier years of roll out *Some AGYW were benefitting but others were not so to equalise this, the programme changed who received which incentive | *Cash transfers were replaced by vocational training *AGYW receiving start-up capital stopped receiving cash transfers | *Unintended outcome was that many AGYW stopped attending safe spaces because (1) they had not been told about this change in advance and (2) some AGYW ended up not receiving both cash transfer or the startup capital (G 2018) | *Gem 2018 (those getting startup capital stopped getting CT) *Gem 2019 (CT replaced with vocational) |
|  | *As part of DREAMS, some AGYW were selected to receive additional benefits or incentives and some were not | *AGYW used to receive cash transfers and start-up kitty as part of intervention in the earlier years of roll out | *AGYW stopped receiving the business start-up kitty and cash transfers *AGYW parents were told by the implementing offices that cash transfers had been stopped because the mentor was away (i.e., incorrectly blaming the mentor) but then the mentor came back and the AGYW had stopped attending safe spaces (V-19) | *DREAMS programme decision | *Viwandani (2018, 2019) |
|  | *AGYW used to receive school fees subsidies as part of the intervention in the earlier years of roll out | *The school fees subsidy was removed from the programme leading to AGYW leaving the programme | *Dignity packs given to AGYW instead of school fees (to compensate for school fees removal?) | *To encourage AGYW to rejoin/stay in the program and start attending safe spaces again | *Gem 2019 |
|  | *AGYW used to receive cash transfers as part of the intervention in the earlier years of roll out | *Cash transfers stopped being included in the intervention | *Mentors encouraged AGYW to rejoin safe spaces when cash transfers stopped *They explained why some AGYW got more *Ran skills training in safe spaces to show that it was okay to not get the cash transfers, there were other benefits and solutions | *To help solve the AGYWs issues and concerns *To ensure that the safe spaces remained a safe space | *Gem 2018 |
|  | *New component of intervention | *Originally My Health My Choice was taught to AGYW but was mostly benefitting 15–17-year-olds | *SHUGA introduced as a new programme intervention to target AGYW aged 17-24 years *SHUGA taught AGYW about HIV infection, prevention etc. | *To benefit AGYW above 17 years as well | *Gem 2019 |
|  | *New component of intervention |  | *LEAP introduced as a new strategy within HTS intervention | *To reduce HIV infection / control the epidemic | *Viwandani 2019 |
|  | *New component of intervention |  | *Partner notifications introduced as a programme intervention |  | *Korogocho 2019 |
|  | *New component of intervention | *AGYW used to be enrolled at age 10 years | *Started enrolling AGYW at age 9 years | *To maximise cover / reach of the DREAMS programme | *Korogocho 2019 |
|  | *New component of intervention |  | *Introduced PNS and HIV self-test, including for partners of AGYW | *To increase partner testing | *Korogocho 2019 |
|  | *New component of intervention | *SASA (Start Awareness Support Action) | *MAP replaced SASA as a strategy (not a different intervention, just a different strategy for the same intervention) | *To reduce GBV in the community | *Viwandani 2019 |
|  | *New component of intervention | *Specific age groups of AGYW were enrolled into the programme | *Ages of in-school girls expanded: the 20-24 category was subsumed into 18-24, and the 15-19 category became 15-17 |  | *Viwandani 2017 |
|  | *Intervention implementation was led by local partners (IP) | *Implementation was led by different implementing partners (IP) in the different locations | *IP variation in interventions: KCCB and IRDO doing things differently (but within Gem) |  | *Gem 2018 |
|  | *Manual/curriculum designed for the safe spaces that mentors could (/were meant to) follow/teach | *Mentors reported that the manual was very heavy and big, and difficult to carry | *Some mentors stopped carrying the manual *Some mentors took notes from the manual and took those to the safe spaces instead | *To avoid carrying the heavy manual, make things easier for mentors | *Viwandani |
|  | *Manual/curriculum designed for the safe spaces that mentors could (/were meant to) follow/teach | *Manual content not relevant *Manual content not revised *Manual not disability friendly or friendly to HIV+ AGYW *Repetitive material that bores AGYW, leading to lower attendance *Manual not broken down for specific age groups *Mentors have difficulty delivering material to all age groups | *Some mentors made changes or adaptations to the curriculum/manual that they used in the safe spaces *Usually due to non-applicability of the manual for certain AGYW ages in some cases | *To ensure alignment of content with AGYW ages? | *Viwandani *Korogocho |
|  | *As part of the DREAMS programme, AGYW were eligible for referrals and access to different health and SRH service providers | *One service provider would visit the AGYW at one time | *Mentors/IPs worked with the service providers so that multiple service providers visited the AGYW at the same time (one appointment instead of multiple appointments) | *To increase efficiency and ensure AGYW received as many services as possible | *Gem 2018 |
|  | *Vocational trainings were scheduled for AGYW as part of the DREAMS programme | *The vocational schools and courses were limited | *Mentors / IPs provided capacity building on combination socio-economic approaches |  | Korogocho |
|  | *ABYM were not part of the original DREAMS programme / intervention | *Not including ABYM was an issue that came up a lot *Implementers and mentors shared that excluding ABYM led to familial dysfunction and ABYM feeling left out | *Sensitizing partners *Partner testing *Some activities with ABYM |  | *Gem 2019 *Viwandani *Korogocho |
| **2** | **CFIR domain: Outer setting - Factors external to the implementation team** | | | | |
| **2A** | **Community perspectives** | | | | |
|  | *PrEP offered in communities, and to DREAMS participants as part of programme implementation | *Lack of knowledge, misconceptions or stigma about PrEP *Reluctance to take PrEP | *Additional and increased reactive community sensitization about PrEP *Enhanced adherence counselling for AGYW *Giving out IEC materials (V-19) *Bringing in a different organisation to do PrEP advocacy (G-19)  *Utilising the radio for community sensitization and information provision *Making AGYW PrEP ambassadors *Training service providers to disseminate information (K) | *Reduce stigma *De-mystify myths *Helping people reduce their risks *Increase programme acceptance among AGYW and the community | *Viwandani 2019 *Gem 2019 *Korogocho |
|  | *Roll out of DREAMS interventions | *Community unacceptance about DREAMS interventions due to community beliefs that DREAMS were 'illuminati' | *Community sensitization: illuminati and other concerns talked through by IPs and mentors *Community forums held in Gem | *Increase programme acceptance in the community | *Gem 2018, 2019 (2019 reports: community is happy now) *Korogocho 2019 (2019 reports: programme still not fully accepted) |
|  | *Community hesitations and misconceptions about DREAMS | *Community perceives DREAMS negatively and doesn't accept interventions *A clip aired on citizen TV that made this worse | *Increased community sensitization efforts in response to the clip that was aired on Citizen TV to spread information about DREAMS *Hotline | *Increase programme acceptance in the community *Damage control due to misconceptions after the clip aired on TV | *Korogocho |
|  | *Roll out of DREAMS interventions | *Mentors and IPs felt valued and comfortable meeting with parents, teachers and community members due to high acceptance in the community | *Mentors and IPs followed up with AGYW, their parents and teachers to discuss progress of AGYW and understand why they weren't attending safe spaces | *Increase/encourage attendance of AGYW at safe spaces | *Gem 2019 |
|  | *Safe spaces and mentorship meant as support for AGYW, but some parents not letting their children attend | *Parents have high expectations of DREAMS *Parents discouraged and spread misinformation when AGYW don't receive cash transfers *Sometimes parents disapprove of DREAMS content (K & V)  *Some parents don't let AGYW attend safe spaces (K) | *IPs set up a program advisory committee to get feedback *Mentors and IPs have extra meetings with parents *IPs set up feedback boxes *Community radio *Talking to parents during other interventions (K) | *Increase/encourage attendance of AGYW at safe spaces *Increase programme acceptance among parents | *Viwandani *Korogocho |
| **2B** | **Setting** | | | | |
|  | *Long travel distances between AGYW homes, safe spaces, vocational schools, service delivery centres | *Distance made intervention delivery difficult and tiring for mentors *Disincentivised AGYW from safe space and vocational school/training attendance | *HTS counsellors were invited into safe spaces, rather than referring AGYW to go to them (G-19) | *Increase/encourage attendance of AGYW at safe spaces *Increase programme acceptance among AGYW | *Gem 2019 |
|  | *Electricity shortages | *Makes intervention delivery difficult for mentors / IPs | *Hired a TV and battery packs to show SHUGA | *Improve programme delivery | *Gem 2019 |
|  | *Long travel distances between AGYW homes and safe spaces | *Distance to safe space locations too long, along with disturbances in the area *AGYW requested splitting safe space into two groups for different areas (e.g. Olengo) | *Safe space location changed *Safe spaces split into multiple groups | *Increase/encourage attendance of AGYW at safe spaces | *Gem |
| **3** | **CFIR domain: Inner setting - Attributes of the implementing organisations** | | | |  |
| **3A** | **Resources** | | | |  |
|  | *Issues with intervention resources | *At times, shortage of resources needed for the intervention, such as learning materials, negatively impacted intervention delivery | *Lack of learning materials or resources needed for safe space activities. Mentors adapted with different materials: using flip charts instead of notebooks when no notebooks were provided *Some mentors made faux footballs using polythene bags when AGYW wanted to play football but no balls were provided, or balls were shared between groups *'Enrichment programs' for AGYW *Some mentors had to adapt where they ran the safe space sessions, for example when the classrooms assigned to them were not free they conducted the safe space in the school playground, but this didn't work well | *Increase/encourage attendance of AGYW at safe spaces *To motivate AGYW *To teach AGYW skills and boost their existing talents - leading to some AGYW being hired *To improve programme delivery | *Gem 2018 *Korogocho 2018 |
|  | *Issues with staff funding | *Lack of or reduced, mentor funding *Some mentors were laid off meant some mentors had to lead more than one safe space *Led to extra workload for mentors still working for DREAMS *One mentor ended up running a mentorship program for two different schools and couldn't manage it effectively | *Mentor forums to discuss problems *Regular mentor meetings | *To help find solutions to problems *To check each other’s progress | *Gem 2019 *Gem 2018 (one mentor for two schools) |
|  | *Issues with staff funding | *Lack of, or reduced, funding for mentors *Some mentors were laid off | *Some mentors merged two safe spaces into one due to lack of mentors  *This had an unintended negative result: increased travel distance to safe spaces for AGYW and mentors, decreased AGYW attendance *Mentors initiated creative activities such as soap making, ball games, singing, dancing | *Increase/encourage attendance of AGYW at safe spaces *To ensure that the AGYW whose mentors were laid off still had access to safe spaces and mentorship | *Gem 2018 (days/times of safe spaces) *Gem 2019 (merging safe spaces) |
|  | *Issues with intervention funding | *Insufficient meeting spaces for safe spaces and other activities *Reduced meeting time with AGYW *Unable to provide all DREAMS programme activities in the office | *Led to mentors changing safe space locations but this confused AGYW and led to reduced attendance *Implementing partners partnered with other (non-DREAMS) organisations to utilise their space for DREAMS activities | *To improve programme delivery *Increase/encourage attendance of AGYW at safe spaces | *Korogocho |
| **3B** | **Internal communications** | | | |  |
|  | *Mentors unsure of their position in relation to AGYW and programme decision-makers | *Some mentors felt they were stuck in the middle of AGYW/parents and implementation offices which caused conflict - e.g., stopping cash transfers was not mentor level decision, but instead of telling AGYW they didn't qualify for the intervention the office told them they didn't receive it because the mentors didn’t pass on AGYW names to the office |  |  | *Viwandani 2018 |
|  | *Hierarchy among programme decision-makers | *School fees subsidies were decided through application letters and validation | *Mentors tried for needy AGYW to receive school fees subsidies / incentives, but they felt they were not listened to by their seniors in the programme | *For needy AGYW to not drop out of the DREAMS programme by providing them the support they needed | *Gem 2019 |
| **4** | **CFIR domain: Individuals Roles and Characteristics** | | | |  |
| **4A** | **Individual circumstances of AGYW (construct: intervention recipients)** | | | | |
|  | *Safe spaces and mentorship meant as support for AGYW | *Older AGYW not attending SS *Conflict between AGYW and staff *Power dynamics between mentors and AGYW, AGYW don't listen to mentors | *Reporting AGYW to the 'office' or higher-ups to increase their attendance *Particularly an issue with the 20-24 year olds who 'threaten you' if you are 'polite', so have to tell them they will not get the CT anymore (K-17) *Mentor tries to talk with AGYW, escalates issue to supervisor if necessary (V-) *Separate safe space groups so that married AGYW are matched with married mentor (G-) | *Increase/encourage attendance of AGYW at safe spaces *Increase/encourage attendance of AGYW at vocational schools/training *Improve programme acceptance among AGYW | *Korogocho (2017, others) *Viwandani |
|  | *Safe spaces and mentorship meant as support for AGYW | *15–19-year-old AGYW busy with education, which takes precedence leading to low programme attendance | *Mentors rescheduled safe space for weekends | *Increase/encourage attendance of AGYW at safe spaces | *Korogocho |
|  | *Safe spaces and mentorship meant as support for AGYW | *Troubled AGYW sought mentors outside of safe spaces to speak to them about personal problems | *Mentors counselled AGYW to help solve their problems | *Improve AGYW wellbeing *Efficient running of safe spaces | *Gem 2018 |
|  | *Safe spaces and mentorship meant as support for AGYW  *Vocational trainings scheduled for AGYW | *Young women with children and partners have competing priorities and low attendance at safe spaces and vocational training (all 3 locations) *Some AGYW receive resistance or backlash from partners for attending (K & V). *Some AGYW expect incentives to attend safe spaces such as cash transfer (K) | *Mentors initiated creative activities such as soap making, ball games, singing, dancing at the safe spaces *Mentors scheduled one-on-one meetings with AGYW and partners, if necessary, to discuss private matters *Mentors invited AGYW to their homes during winter break from school (G-19) *Mentors implemented activities such as 'table banking' at safe spaces *Mentors followed up with AGYW and their partners including on weekends; | *Increase/encourage attendance of AGYW at safe spaces *Increase programme acceptance, among AGYW and their partners *Comfort AGYW | *Gem 2019 *Viwandani *Korogocho |
|  | *Safe spaces take married girls away from home  *ABYM not included in interventions | *AGYW's spouses causing issues with safe space attendance | *Mentors spoke with AGYW's spouses to allow them to attend safe spaces | *Increase/encourage attendance of AGYW at safe spaces | *Viwandani 2017 |
|  | *Vocational trainings scheduled for AGYW | *AGYW who had children could not attend vocational schools / training due to lack of childcare | *Implementing partners (IPs) asked vocational schools to offer baby-sitting so AGYW could attend | *Increase/encourage attendance of AGYW at safe spaces *Increase/encourage attendance for vocational schools/training | *Viwandani 2019 |
|  | *Vocational trainings scheduled for AGYW | *Lack of attendance at vocational trainings | *Implementing partners (IPs) did extra follow-ups with AGYW who were not attending safe spaces or vocational schools/training to try and encourage them to attend | *Increase/encourage attendance of AGYW at safe spaces | *Korogocho 2019 |
|  | *A curriculum manual was designed for the safe spaces that mentors were meant to follow/teach | *Mentors felt that the manual content was not relevant for all ages of AGYW, and was not broken down for specific age groups | *Mentors adapted safe space curriculum delivery according to AGYW ages (K-17) | *Making intervention content more appropriate for AGYW ages *To improve intervention fit with recipients | *Korogocho 2017 *Viwandani |
|  | *AGYW experience of gender-based violence, abuse | *AGYWs experience of violence, including partner violence, made AGYW vulnerable and in need of services *Partner violence discouraged AGYW from attending intervention programmes (K, V) *Difficulties communicating with communities/tribes (V) *Violence was normalised | *GBV sensitization, including with partners (V-19) *IPs and mentors held targeted conversations with partners of AGYW (G-19) *Mentors met with AGYW and their partners to discuss the benefits of DREAMS (K) | *So that the AGYW had long-term benefits from DREAMS, and had something after DREAMS ended *Increase programme acceptance including from partners *Comfort and protect AGYW *Increase/improve programme access *Increase/encourage attendance of AGYW at safe spaces | *Viwandani (2019, others) *Korogocho *Gem 2019 |
|  | *AGYW experience of gender-based violence, abuse | *AGYW experience of abuse and violence in their homes and within their families made the DREAMS referral for services process difficult *Increased AGYWs need of services | *IPs and mentors provided personal funds for post violence cases (G-19) *IPs and mentors accompanied AGYW to receive services, including visiting the police (V) *If these circumstances were related to the AGYW's families, it could make referrals difficult for IPs and mentors; in this case they sometimes did not refer, and this was an adaptation from the intervention? (K) | *Comfort and protect AGYW *Increase/improve programme access | *Viwandani *Korogocho *Gem 2019 |
|  | *AGYW spent time with boda boda (motorcycle) drivers for various reasons - including out of necessity to travel etc. | *Boda boda guys sometimes had a negative influence on AGYW and spread misinformation about DREAMS | *IPs and mentors had targeted conversations with community chiefs and boda boda drivers (G-19) *Partnerships with other organisations in the community to increase circumcisions and HIV testing (V) | *To protect the AGYW *To increase HIV testing (V) | *Gem 2019 *Viwandani |
|  | *DREAMS aimed to provide AGYW with opportunities and more resources (e.g., funds, uniforms, start-up kits etc.) | *At times, not all AGYW in need received the financial/resource interventions *Some AGYW needed more help, support and resources which they didn't receive | *IPs and mentors helped AGYW apply for government funds, e.g., the Youth Fund, different women's groups *Mentors at times gave AGYW clothes when needed, e.g., when the uniform subsidy ended and one girl had a torn blouse *Some mentors used their own salary to help AGYW, e.g., buying them food *Mentors helped AGYW apply for start-up kits *Many other examples from Viwandani R2!* | *To help AGYW understand the 'financial capability' intervention *To help AGYW actually receive the intervention *To just help AGYW in need when the intervention was not able to | *Gem 2018 (fund apps) *Gem 2019 (clothes) *Viwandani 2017 (own salary, start-up kits) *Viwandani 2018 |
|  | *Trainings scheduled for AGYW as part of the DREAMS intervention by the implementing partners | *Some AGYW were not understanding the content of trainings due to low literacy *Some AGYW felt discouraged and stopped attending safe spaces | *Mentors organised literacy classes for AGYW *Mentors translated programme material into Luo *Mentors taught AGYW literacy skills | *To help AGYW attend vocational training *To improve the uptake of AGYW attending, i.e., improve retention rate rather than enrolment, because AGYW would enrol but then drop out due to childcare issues *Increase/encourage attendance of AGYW at safe spaces | *Gem 2019 |
|  | *Donors had targets for numbers of AGYW attendance of safe spaces | *It was a challenge to meet donor targets because of high migration of in-school AGYW *It was difficult to know if AGYW chose to stop attending safe spaces or if they had just moved out of the community | *Mentors followed up with AGYW to understand the lack of attendance | *Increase/encourage attendance of AGYW at safe spaces | *Gem 2019 |
|  | *Enrolment into programme: losing AGYW but also reality that cannot enrol all AGYW | *AGYW leaving the area or getting married etc. and so leaving the intervention *Some vulnerable AGYW being left out of DREAMS | *Enrolling more AGYW, but focussing on those who were vulnerable and those who were requesting to be enrolled *Implementers kept a list of AGYW who were not enrolled but wanted to be part of the DREAMS programme and then enrolled them when there was space | *To make up the number of participants *To not leave out AGYW who wanted to be part of the program *To improve programme delivery | *Gem 2018 *Viwandani 2019 |
| **4B** | **Attitudes towards/value placed on intervention, belief in own capabilities to execute actions to achieve goals** | | | | |
|  | *Relationship between implementing partners (IPs) and mentors | *IP feel strong organisational support (V: makes work more team oriented and effective) *IPs view mentors very positively and feel motivated and supported to implement the intervention | *IPs created checklist to evaluate each intervention; utilised mentors' help in the screening process of enrolling AGYW (G) *Staff have technical advisory and continuous improvement committees to discuss challenges and solutions *Staff personally fund retreats (V) | *To improve programme delivery | *Gem 2019 *Viwandani |
|  | *DREAMS had a set curriculum that mentors had to teach AGYW in safe spaces | *Some mentors don't approve of topics in the curriculum such as family planning | *Some mentors didn't teach topics that they disagreed with or felt was inappropriate for the AGYW in their safe spaces |  | *Gem 2019 |
|  | *Safe spaces and mentorship meant as support for AGYW | *Some AGYW were unhappy and unmotivated despite the interventions | *Mentors let AGYW choose the activities they wanted to do in the safe spaces *IPs came up with ‘five pillars for SAB (Social Asset Building) programming’ which included making the intervention 'AGYW centric' and 4 others (see quote) | *To motivate AGYW *Increase/encourage attendance of AGYW at safe spaces *Improve social asset building (SAB) uptake | *Viwandani 2019 |
|  | *Safe spaces and mentorship meant as support for AGYW | *Some AGYW were unhappy and unmotivated despite the interventions | *Mentors let the AGYW moderate some of the safe space sessions | *To motivate AGYW *Increase/encourage attendance of AGYW at safe spaces | *Korogocho 2018 |
|  | *Safe spaces and mentorship meant as support for AGYW | *Some AGYW were unhappy and unmotivated despite the interventions | *Mentors introduced new and fun activities in the safe spaces which varied by mentor and safe space - often referred to as the 'mentor's approach' which differed a lot *Adaptations to safe space content included: providing AGYW with certificates, table banking, soap making activities, decorating, catering skills, some mentors paid AGYW for their skills, giving advice to girls, playing games  *Talent shows, making detergent, not always following the manual, asking girls what they want to do (K)  *Ball games (V) | *To motivate AGYW *Increase/encourage attendance of AGYW at safe spaces | *Gem 2019 (mentor's approach) *Gem 2018 (skills development, choosing girls who are not getting other benefits but still coming to safe spaces, giving advice, football, inter debates) *Korogocho 2018 *Viwandani 2018 |
|  | *Safe spaces and mentorship meant as support for AGYW | *Some AGYW were unhappy and unmotivated despite the interventions | *Mentors started 'inter-safe spaces' *Set up football competitions | *To motivate AGYW *Increase/encourage attendance of AGYW at safe spaces | *Gem 2018 *Gem 2019 |
|  | *Safe spaces and mentorship meant as support for AGYW | *Some AGYW were unhappy and unmotivated despite the interventions | *Mentors made AGYW 'buddies' in safe spaces to motivate each other to attend safe spaces together | *To motivate AGYW *Increase/encourage attendance of AGYW at safe spaces | *Viwandani 2017 |
|  | *Safe spaces and mentorship meant as support for AGYW | *Some AGYW were unhappy and unmotivated despite the interventions | *Mentors made home visits to AGYW to encourage safe space attendance *Some mentors went to actually pick up AGYW from their homes to bring to safe spaces | *To motivate AGYW *Increase/encourage attendance of AGYW at safe spaces | *Korogocho 2018 *Viwandani 2017 *Viwandani 2018 |
|  | *Safe spaces and mentorship meant as support for AGYW | *Some AGYW were unhappy and unmotivated despite the interventions | *IPs provided airtime for mentors to call AGYW to encourage attendance, but the provided airtime was not enough | *To motivate AGYW *Increase/encourage attendance of AGYW at safe spaces | *Korogocho 2018 |
|  | *Safe spaces and mentorship meant as support for AGYW | *Some AGYW were unhappy and unmotivated despite the interventions | *Mentors planned a fun event for the AGYW on a weekend using their own money (after asking IPs for money but they didn't give) | *To motivate AGYW *Increase/encourage attendance of AGYW at safe spaces | *Viwandani 2017 |
|  | *Role of mentors in DREAMS | *Some mentors unhappy because excluded from programme meetings and decision making *Some mentors felt rushed and pressured to meet donor targets | *Mentors organised forums to exchange tips and share experiences, 'retreats' and monthly meetings: self-funded meetings to discuss challenges and find solutions *These were sometimes funded by the program but not always | *To make sure the AGYW received the services they needed *To ensure AGYW wellbeing *To improve programme delivery | *Viwandani 2019 *Gem 2018 (mentors met monthly to plan/discuss) |
|  | *PrEP offered in communities, and to DREAMS girls as part of programme implementation | *Some staff (IPs/mentors) themselves held misconceptions about PrEP and were thus spreading misconceptions *Low uptake of PrEP | *IPs/mentors held meetings about PrEP to demystify it amongst themselves | *To improve programme delivery *To improve programme acceptance | *Viwandani |
|  | *AGYW referrals to doctors, clinicians and other specialists was part of the DREAMS programme *This was implemented differently in the three locations | *AGYW in need | *Mentors did referral follow ups (K) *Mentors referred AGYW to doctors for GBV incidents, but many mentors also did follow ups to ensure AGYW were okay, at times paying doctor fees themselves that wasn't covered by the intervention (G) *Location differences: in Gem referrals and follow ups seemed to be mentor-led and paid, but in Korogocho a standard protocol was followed even for follow-ups. Viwandani similar to Korogocho: referrals and follows ups seem to have SOPs depending on which clinic or person being referred to, and even when no paperwork, no issues of payment have been mentioned | *To encourage and retain AGYW in DREAMS  *To ensure the wellbeing of AGYW | *Korogocho 2019  *Gem 2018 *Viwandani 2017 |
|  | *Either pregnancy tests were not meant to be provided as part of the intervention, or they were, and this did not go as planned | *AGYW unable to pay for pregnancy tests | *Mentors provided personal funds to help pay for pregnancy tests *IPs kept pregnancy tests at the office for AGYW to have access to | *Improve AGYW wellbeing *Comfort AGYW *Improve programme access | *Korogocho |
|  | *Unhappy parents of AGYW | *AGYW parents not allowing them to attend safe spaces etc. especially if there were no tangible or visible benefits | *Mentors followed up with AGYW parents to discuss the interventions benefits etc. with them (K)  *IPs spoke with caregivers/parents of AGYW after monetary benefits (cash transfers) were stopped to explain why these had stopped, but that attendance was still important (V) | *Increase/encourage attendance of AGYW at safe spaces *Appease AGYW parents so they would encourage/allow AGYW to attend | *Korogocho 2017 *Viwandani 2019 |
|  | *AGYW enrolment into DREAMS *Attrition of AGYW from programme | *AGYW from inaccessible areas being left out of the programme | *IPs/mentors requested community leaders to enrol AGYW from areas the mentors can't access | *To maximise cover / reach of the DREAMS programme | *Korogocho 2017 |
|  | *Cash transfers were originally part of the DREAMS programme but later removed | *Some AGYW received cash transfers and some did not *AGYW who did not get cash transfers were demotivated and did not attend safe spaces | *Some mentors found ways for AGYW to receive cash transfer - for those who weren't getting any | *Increase/encourage attendance of AGYW at safe spaces *To motivate AGYW *To improve AGYW wellbeing | *Viwandani 2017 |
|  | *Cash transfers were originally part of the DREAMS programme but later removed | *Some AGYW received cash transfers and some did not *AGYW who did not get cash transfers were demotivated and did not attend safe spaces | *Some mentors told AGYW that those who attended safe spaces regularly will receive cash transfers | *Increase/encourage attendance of AGYW at safe spaces |  |
|  | *Education subsidies were part of the original intervention | *Not all AGYW were eligible for or received the education subsidy *AGYW parents felt angry and talked badly to mentors | *IPs put a list of the AGYW who received education subsidies outside of their office to increase transparency in the community | *Increase programme acceptance from community members | *Korogocho |
|  | *Mentors were given 'manuals' that contained the SS curriculum, big books that they could teach from |  | *Mentors didn't carry manuals to the safe spaces for the 20–24-year-olds as this made these AGYW think it's 'like school' and they 'sleep' - so they make notes from the manual instead on what they will teach that day |  | *Viwandani 2018 |
|  |  | *Not enough space for mentors to set up libraries for AGYW | *Mentors donated library books to a partner organisation which allowed AGYW to use that space as a library *Mentors donated textbooks |  | *Viwandani 2019 *Gem 2019 |
| **5** | **CFIR domain: Implementation Process** | | | |  |
| **5A** | **External systems such as donors, governments, previous organisations and schools, that implemented implementation (construct: innovation deliverers)** | | | |  |
|  | *Safe space logistics often relied on schools | *Mentors faced difficulties in scheduling safe space meetings due to issues faced at the schools | *Mentors were flexible about safe space session organising and logistics, for example, some changed their sessions to weekends due to school timings *Some mentors held safe spaces in the playground when classroom wasn't available |  | *Gem 2018 (days/times of safe spaces) *Gem 2019 |
|  | *Health facilities were supposed to provide AGYW with necessary services | *Limited resources at health facilities and limited clinicians from MOH *Increased workload of implementers and mentors *Strong links with health facilities *Smooth referral process | *Mentors/IPs used personal funds to pay for emergency contraceptives *Mentors and IPs were trained in continuous medical education and delivered biomedical talks instead of clinicians *Reduced HTS testing to once a year instead of every 3 months | *Increase programme access *Improve programme delivery | *Gem 2019 |
|  | *Past organisations had run interventions / similar programmes in the communities but did not deliver on their promises | *Communities had difficulty trusting DREAMS due to previous bad experiences *Communities expected to receive cash or other financial incentives to participate in programme | *Mentors and IPs increased sensitization with communities in reaction to these issues | *Increase programme acceptance | *Viwandani *Korogocho (no adaptation identified I think, problem reported though) |
|  | *Government bureaucracy | *Government delays in issuing birth certificates led to delayed issuing of DREAMS identification numbers (IDs) | *Mentors (and IPs?) allowed unenrolled AGYW to attend safe spaces | *Increase programme access *Improve programme delivery | *Gem 2019 |
|  | *Government facilitation | *A Memorandum of Understanding (MOU) between DREAMS implementing organisations and government health facilities | *Could the MOU be a context-specific understanding? Was this occurring in all DREAMS locations? | *Allowing AGYW to have easier access to health facilities | *Viwandani |
